# Automated Literature Screening for Hepatocellular Carcinoma Treatment Through Integration of 3 Large Language Models: Methodological Study

**DOI:** 10.2196/76252

**Published:** 2025-09-08

**Authors:** Chen Pan, Wei Lu, Bingliang Chen, Gang Zhang, Zhiming Yang, Jingcheng Hao

**Affiliations:** 1 Department of Hepatobiliary and Vascular Surgery First Affiliated Hospital of Chengdu Medical College Chengdu China

**Keywords:** methodology, large language model, hepatocellular carcinoma, treatment, literature screening

## Abstract

**Background:**

Primary liver cancer, particularly hepatocellular carcinoma (HCC), poses significant clinical challenges due to late-stage diagnosis, tumor heterogeneity, and rapidly evolving therapeutic strategies. While systematic reviews and meta-analyses are essential for updating clinical guidelines, their labor-intensive nature limits timely evidence synthesis.

**Objective:**

This study proposes an automated literature screening workflow powered by large language models (LLMs) to accelerate evidence synthesis for HCC treatment guidelines.

**Methods:**

We developed a tripartite LLM framework integrating Doubao-1.5-pro-32k, Deepseek-v3, and DeepSeek-R1-Distill-Qwen-7B to simulate collaborative decision-making for study inclusion and exclusion. The system was evaluated across 9 reconstructed datasets derived from published HCC meta-analyses, with performance assessed using accuracy, agreement metrics (κ and prevalence-adjusted bias-adjusted κ), recall, precision, *F*_1_-scores, and computational efficiency parameters (processing time and cost).

**Results:**

The framework demonstrated good performance, with a weighted accuracy of 0.96 and substantial agreement (prevalence-adjusted bias-adjusted κ=0.91), achieving high weighted recall (0.90) but modest weighted precision (0.15) and *F*_1_-scores (0.22). Computational efficiency varied across datasets (processing time: 248-5850 s; cost: US $0.14-$3.68 per dataset).

**Conclusions:**

This LLM-driven approach shows promise for accelerating evidence synthesis in HCC care by reducing screening time while maintaining methodological rigor. Key limitations related to clinical context sensitivity and error propagation highlight the need for reinforcement learning integration and domain-specific fine-tuning. LLM agent architectures with reinforcement learning offer a practical path for streamlining guideline updates, though further optimization is needed to improve specialization and reliability in complex clinical settings.

## Introduction

### Background

Primary liver cancer (PLC) is the sixth most commonly diagnosed cancer and the third leading cause of cancer-related deaths worldwide [1]. Among PLCs, hepatocellular carcinoma (HCC) is the most common, accounting for approximately 80% to 90% of liver cancer cases worldwide [2,3]. HCC has a poor prognosis. In general, the overall 5-year survival rate for patients diagnosed with HCC is typically <20%, which is similar to that for lung cancer but notably lower than for colon cancer [4-6]. In addition, the treatment of HCC is highly individualized and complex [7]. In general, for patients with early-stage HCC, surgical intervention is a viable option, and they often have a relatively good prognosis [8]. However, patients with HCC frequently present at an intermediate or advanced stage, and surgery alone may not be beneficial. Conversion therapies, guided by systemic and local therapy approaches, have demonstrated efficacy in downstaging and potentially resecting intermediate and advanced HCC, as well as enhancing outcomes [9,10]. It is evident that HCC is notably more complex to treat than many other medical conditions, requiring a nuanced decision-making process guided by evidence-based research and clinical guidelines.

However, there is often a delay between research publication and its incorporation into academic discourse, especially with guidelines based on systematic reviews and meta-analyses. An example of this lag can be observed in the 2021 guidelines disseminated by the Society for Immunotherapy of Cancer, which did not incorporate the outcomes of the HIMALAYA trial that same year [11,12]. This randomized controlled trial (RCT) demonstrated that the single tremelimumab regular interval durvalumab regimen, a combination of a single high dose of tremelimumab given as a first-line treatment followed by maintenance treatment with durvalumab, significantly improved overall survival and constitutes a new treatment option [12]. As such, the timely updating of information is of paramount importance, as it facilitates the refinement of guidelines and the evolution of treatment plans. Systematic reviews and meta-analyses represent the highest levels of evidence in the hierarchy of evidence [13]. They play an important role in updating the guidelines. However, meta-analysis and systematic reviews are time-consuming processes, especially the process of collecting and screening literature records [14]. To keep guidelines up to date, faster methods for conducting meta-analyses are essential. Given the standardized PRISMA (Preferred Reporting Items for Systematic Reviews and Meta-Analyses) framework, integrating artificial intelligence (AI) can help automate and improve this process.

AI is a discipline that allows computers to simulate intelligent human behavior, enabling machines to emulate human intelligence and problem-solving abilities [15]. Large language models (LLMs), built on transformer architectures, have become a central focus in AI research. By predicting the next word in a sequence, LLMs learn statistical patterns in text. Leveraging the transformer’s self-attention mechanism, they effectively capture long-range dependencies and contextual meaning [16]. Therefore, LLM can be used to extract structured data from unstructured texts [17,18]. Previous studies have explored the automation of the literature screening process in systematic reviews and meta-analyses using a single LLM with good results [19,20]. However, previous studies often used a single LLM without simulating collaboration among models in different roles [19,20]. Meanwhile, newer models with more parameters or reinforcement learning have shown better performance than earlier approaches.

### Objectives

In this study, we propose a literature screening workflow that leverages 3 distinct language models to automatically analyze titles and abstracts from multiple databases, aiming to support systematic reviews and meta-analyses. We evaluate its effectiveness in simulating real-world screening procedures and conflict resolution, using datasets from published studies. Furthermore, we investigate the types and causes of model errors across different datasets and explore the integration of reinforcement learning to enhance decision-making.

## Methods

### Overview

The overall process of this study is divided into 3 stages: application design, validation, and statistical analysis. In the application design section, a multimodule system was developed to parse literature entries, interface with the LLM service provided by the cloud computing platform, and perform literature entry analysis. Verification involved conducting simulations and constructing datasets based on meta-analyses published in high-impact journals, followed by comprehensive validation through analytic procedures implemented in our previously developed system. The final phase involved a comparative and analytic examination of the results generated by the system and the definitive inclusion outcomes of the aforementioned meta-analyses. All programmatic flows were implemented in Python (version 3.12.3; Python Software Foundation) and R (version 4.4.1; R Foundation for Statistical Computing).

### Application Design

**Figure 1:**
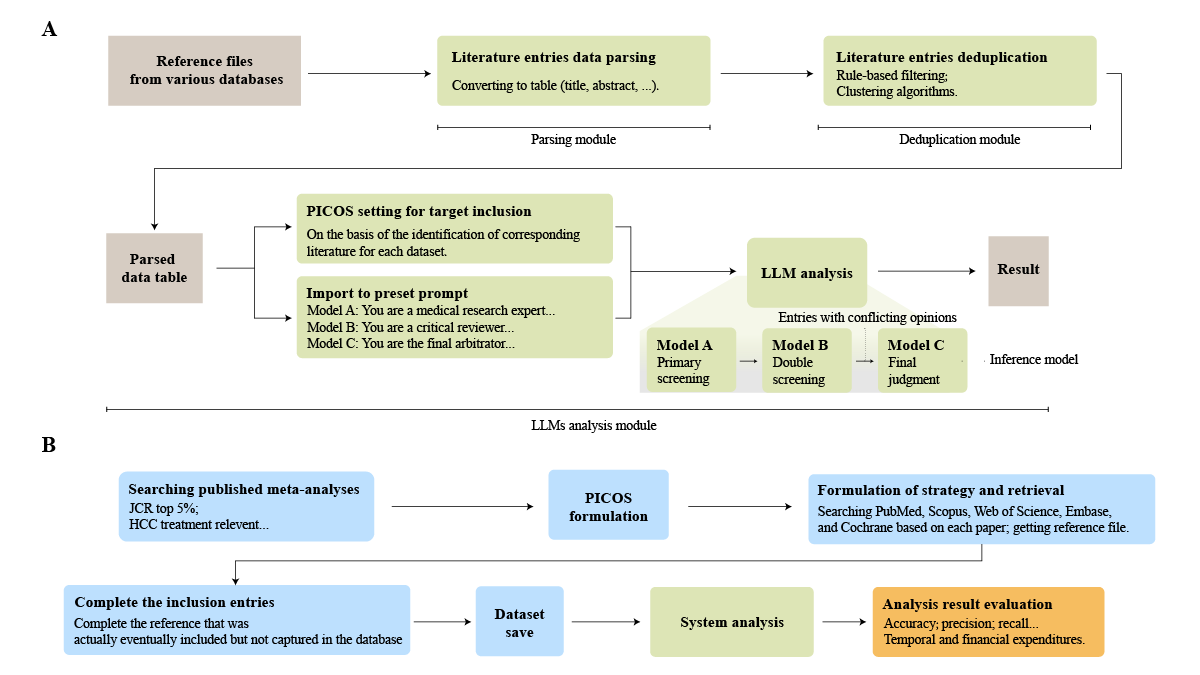
Schematic workflow of the study. (A) Workflow of the developed system. The system first parsed the fields in the referenced documents, then used the deduplication function to remove duplicates and parsed a data table. Subsequently, after setting the PICOS objectives of the studies to be included, it was sequentially submitted to three large language models for processing in combination with the prompts. (B) Workflow of the validation process performed for the system. The verification step first relied on existing meta-analyses in highly cited journals, manually identified their PICOS, and reproduced them according to the methods described in the articles to construct a dataset. Subsequently, the analysis process was carried out using the system we had developed. HCC: hepatocellular carcinoma; JCR: Journal Citation Reports; LLM: large language model; PICOS: Population, Intervention, Comparison, Outcome and Study Design.

This study constructed a multimodule system consisting of 3 major parts: literature entry data parsing, duplicate item removal, and LLMs analysis. The workflow of the developed system is shown in Figure 1A. In the literature record parsing part, automated programs were used to process search results saved in RIS and NBIB formats, extracting key fields such as titles, author information, and abstracts into structured data tables. The deduplication part deployed a process based on certain fields of the literature entries to remove highly duplicated entries, as detailed in Multimedia Appendix 1. In the analysis of LLMs part, the program preset roles and inserted the population, intervention, comparison, outcome, and study design (PICOS) guidelines for 3 different large models and embedded the literature entries that needed to be analyzed into prompts in batches. Any analysis task performed by the system must contain at least 1 nonempty element from PICOS. Tasks lacking specific PICOS elements were flagged by the program as unspecified and triggered an expanded search scope. Specifically, this process was divided into 3 parts. The program first assigned model A as the initial reviewer, which made preliminary inclusion or exclusion judgments on the inputted literature entries based on the predefined PICOS criteria, along with the corresponding reasoning. Subsequently, model B was designated as the critic, reviewing the same literature entries and evaluating model A’s decisions based on the same PICOS criteria, providing its own judgments and reasoning. Finally, model C was assigned the role of arbitrator, reanalyzing those entries for which models A and B had provided conflicting decisions and making the final determination along with the supporting reasoning. The role prompts and the hyperparameters used for these 3 models are provided in Multimedia Appendix 1. In addition, a detailed explanation of this system is provided in Multimedia Appendix 1.

### Validation

The validation section includes the preparation of the verification datasets, system analysis, and data statistics, as shown in Figure 1B. To construct multiple validation datasets, we searched for meta-analyses related to the treatment strategies for PLC. The following inclusion criteria were used: (1) studies published within the past 5 years, (2) meta-analyses of treatment strategies for PLC, (3) studies that reported the search platforms used and described the basic search strategy in the text, and (4) studies published in the top 5% of journals indexed in the Science Citation Index Expanded and compliant with the PRISMA guidelines. The exclusion criteria included studies with search strategies that could not be replicated based on the description in the text, non-English studies, studies without accessible full texts, and studies that overlapped with or duplicated other publications.

After determining the meta-analyses to be included, each meta-analysis was manually reviewed, and the following tasks were undertaken: (1) identifying the PICOS elements by human (leave blank if PICOS components cannot be identified), (2) implementing search strategy to retrieve records according to the method part of each meta-analysis, (3) downloading NBIB or RIS files from various databases, and (4) deduplicating and converting NBIB or RIS files into data tables using the analysis system to construct the datasets. The initial number of candidate records in the data tables was aligned as closely as possible with the corresponding numbers in the PRISMA flow diagrams of the respective meta-analyses. To ensure minimal human intervention in the simulation, any studies that were ultimately included in the original meta-analyses but were missing from the search results were not added back to the datasets.

After constructing all the datasets, 3 major language models were selected based on considerations of research cost and model performance. These models were Doubao-1.5-pro-32k from ByteDance (model A), DeepSeek-v3 (model B), and DeepSeek-R1-Distill-Qwen-7B (model C), with model C distinguished as a reasoning-optimized LLM. The technical specifications for these models are outlined in Table 1. All datasets were analyzed using the previously described analytic workflow of our system, using default hyperparameters as detailed in Multimedia Appendix 1, and the results were exported in tabular format.

**Table 1:** Large language models used in the validation process of the review screening workflow.

Model designation	Model name	Model type	Strengths	Limitations
Model A	Doubao-1.5-pro-32k	Closed; general purpose; MoE^a^	High performance and multimodal capability	Closed source model with low transparency
Model B	DeepSeek-V3	Open; general purpose; MoE	Open source, efficient training model	Signs of distillation may lead to identity inconsistency or output bias
Model C	DeepSeek-R1-Distill-Qwen-7B	Open; reasoning optimized	Open source, reasoning, small and efficient for local deployment	Relatively weak in long-context or multimodal tasks

^a^MoE: mixture of experts.

### Statistical Analysis

The result tables from each validation set analyzed by the system were compared with the actual final results (excluding entries with missing or empty abstracts from the simulated search) included in their corresponding papers. The accuracy, precision, recall, *F*_1_-score, κ, and prevalence-adjusted bias-adjusted κ (PABAK) of the analysis results for all datasets were calculated, where precision and recall were calculated separately based on exclusion and inclusion as the final goal. To improve comparability in imbalanced scenarios, F-β (a weight harmonic mean of precision and recall), balanced accuracy, and Matthews correlation coefficient (MCC) were also evaluated. The statistical indicators of the average were reported using the mean and, where applicable, the standard deviation (SD). The calculation formulas for the statistical metrics are presented in Multimedia Appendix 1. To further evaluate the effect of class imbalance on agreement measures, paired statistical significance tests were conducted to compare κ and PABAK to determine whether κ was systematically depressed under imbalanced class distributions. Depending on the distribution of the paired differences, either a paired 2-tailed *t* test or a Wilcoxon signed rank test was used at a significance level of *P*<.05. In addition, we evaluated the time and economic costs for the analysis of each dataset. The time cost was determined by recording the start and end time stamps from the analysis logs for each dataset. Due to billing latency in cloud computing platforms, the economic cost was estimated through a simulation program based on platform pricing and then validated against actual billing statements. The detailed design of the simulation program is described in Multimedia Appendix 1.

## Results

### Overview of Datasets

On the basis of the simulation process in the validation, 9 meta-analyses were ultimately included and used to construct the simulated datasets [21-29]. These studies were published in various prestigious journals, including the *British Journal of Surgery*, *Liver Cancer*, *Journal of Hepatology*, *JAMA Oncology*, and *Annals of Surgery*. Published between 2020 and 2025, these meta-analyses formed the foundation of the dataset and addressed a diverse range of topics related to PLC treatments, such as systemic therapies, radiation-based approaches, and posttransplant outcomes. These studies, conducted across multiple regions, including the United States, Europe, and Asia, involved single-center and multicenter collaborations. In addition, they used diverse data sources (RCTs, observational cohorts, and individual patient-level data) and applied meta-analytic methods, including pairwise comparisons, network meta-analyses, single-arm analyses, and individual patient data assessments.

In each of the meta-analyses included, 9 datasets were created to represent the initial records identified during their search process, as shown in Table 2. The number of initially identified records in these simulated datasets ranged from 309 to 8733. These datasets encompassed multiple perspectives on the treatment of PLC, including a diverse range of candidate study types, such as RCTs; observational studies (eg, cohort and case-control designs); systematic reviews; meta-analyses; and other evidence-based reports sourced from databases such as PubMed, Cochrane Library, and clinical trial registries. These datasets also featured various types of meta-analyses and varying numbers of initially identified records, reflective of real-world meta-analysis scenarios where data availability and completeness can differ widely. Information on these datasets, including the PICOS criteria corresponding to each meta-analysis and the search strategies used to construct the respective datasets, is provided in Multimedia Appendix 1.

**Table 2:** Characteristics of meta-analysis studies included in the validation stage.

Dataset name	Study title	Included records (n/N, %)^a^	Published journal	Study type
LORC-CH^b^	Laparoscopic versus open resection of hepatocellular carcinoma in patients with cirrhosis: meta-analysis [21]	11/770 (1.4%)	*British Journal of Surgery*	Meta-analysis of 2 interventions based on RCTs^c^ and PSM^d^ studies
FLSTN-AH^e^	First-line systemic therapies for advanced hepatocellular carcinoma: a systematic review and patient-level network meta-analysis [22]	11/5417 (0.20%)	*Liver Cancer*	Network meta-analysis based on RCTs
TLP-AH^f^	Efficacy and safety of transarterial chemoembolization combined with lenvatinib and PD-1 inhibitor in the treatment of advanced hepatocellular carcinoma: a meta-analysis [23]	12/309 (3.9%)	*Pharmacology & Therapeutics*	Single-intervention, multiple-control meta-analysis based on cohort studies
NCN-UH^g^	Comparative efficacy of novel combination strategies for unresectable hepatocellular carcinoma: a network metanalysis of phase III trials [24]	9/1698 (0.54%)	*European Journal of Cancer*	Network meta-analysis based on RCTs
FSTN-AH^h^	Efficacy and safety of frontline systemic therapy for advanced HCC: a network meta-analysis of landmark phase III trials [25]	15/8733 (0.17%)	*JHEP Reports*	Network meta-analysis based on RCTs
PTICI-PTHO^i^	Impact of pre-transplant immune checkpoint inhibitor use on post-transplant outcomes in HCC: a systematic review and individual patient data meta-analysis [26]	21/558 (3.8%)	*Journal of Hepatology*	Individual patient data meta-analysis
SBRT-HMG^j^	Stereotactic body radiation therapy for hepatocellular carcinoma: meta-analysis and international stereotactic radiosurgery society practice guidelines [27]	13/897 (1.4%)	*International Journal of Radiation Oncology - Biology - Physics*	Single-arm meta-analysis based on observational studies
STSN-AH^k^	Systemic therapy and sequencing options in advanced hepatocellular carcinoma: a systematic review and network meta-analysis [28]	13/3522 (0.37%)	*JAMA Oncology*	Network meta-analysis based on RCTs
LRAMC^l^	Liver resection versus local ablation therapies for hepatocellular carcinoma within the Milan criteria: a systematic review and meta-analysis [29]	24/6078 (0.39%)	*Annals of Surgery*	Meta-analysis of 2 interventions based on RCTs and NRTs^m^

^a^N represents the sample size of the corresponding dataset by simulating the retrieval strategy from the corresponding study, followed by system parsing, deduplication, and removal of entries with blank abstracts in our research; n indicates the actual number of final included literature records after excluding those not retrieved by our simulated search strategy and entries with missing abstracts, based on the final inclusion results from the corresponding study.

^b^LORC-CH: laparoscopic versus open resection in cirrhotic hepatocellular carcinoma

^c^RCT: randomized controlled trial.

^d^PSM: propensity score matching.

^e^FLSTN-AH: first-line systemic therapy network meta-analysis for advanced hepatocellular carcinoma.

^f^TLP-AH: transarterial chemoembolization plus lenvatinib plus programmed death receptor 1 inhibitor in advanced hepatocellular carcinoma.

^g^NCN-UH: novel combination network meta-analysis for unresectable hepatocellular carcinoma.

^h^FSTN-AH: frontline systemic therapy network meta-analysis for advanced hepatocellular carcinoma.

^i^PTICI-PTHO: pre-transplant immune checkpoint inhibitors impact on post-transplant hepatocellular carcinoma outcomes.

^j^SBRT-HMG: stereotactic body radiation therapy in hepatocellular carcinoma meta-analysis and guidelines.

^k^STSN-AH: systemic treatment sequencing network meta-analysis for advanced hepatocellular carcinoma.

^l^LRAMC: liver resection versus ablation within Milan criteria.

^m^NRT: nonrandomized trial.

### Performance

**Figure 2:**
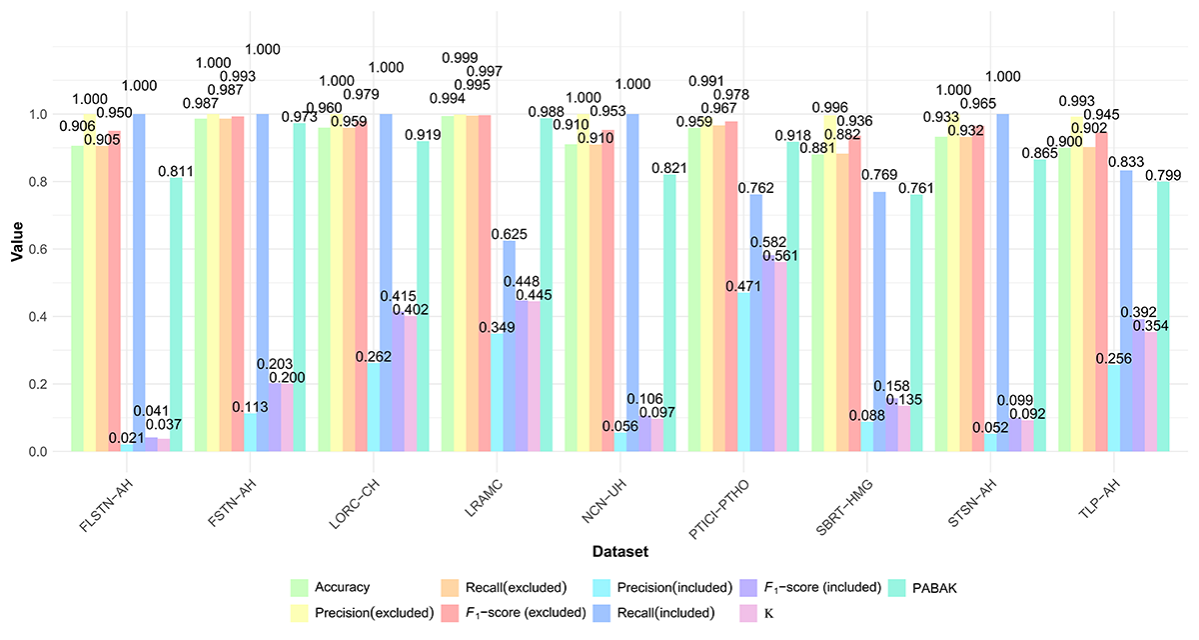
Comparative performance of 3 large language model–based analytic systems across 9 benchmark datasets. The metrics include accuracy, precision, recall, F1-score, κ, and prevalence-adjusted bias-adjusted kappa (PABAK). Precision, recall, and F1-score were calculated twice, once with “included” as the positive class (targeting the correct identification of articles to include) and once with “excluded” as the positive class (targeting the correct identification of articles to exclude). The system achieved high accuracy across all datasets, with recall and PABAK consistently displaying relatively high values, especially when focusing on inclusion. FLSTN-AH: first-line systemic therapy network meta-analysis for advanced hepatocellular carcinoma; FSTN-AH: frontline systemic therapy network meta-analysis for advanced hepatocellular carcinoma; LORC-CH: laparoscopic versus open resection in cirrhotic hepatocellular carcinoma; LRAMC: liver resection versus ablation within Milan criteria; NCN-UH: novel combination network meta-analysis for unresectable hepatocellular carcinoma; PTICI-PTHO: pre-transplant immune checkpoint inhibitors impact on post-transplant hepatocellular carcinoma outcomes; SBRT-HMG: stereotactic body radiation therapy in hepatocellular carcinoma meta-analysis and guidelines; STSN-AH: systemic treatment sequencing network meta-analysis for advanced hepatocellular carcinoma; TLP-AH: transarterial chemoembolization plus lenvatinib plus programmed death receptor 1 inhibitor in advanced hepatocellular carcinoma.

The system performed well in both inclusion and exclusion tasks, as shown in Figure 2. Overall, the system correctly classified 26,735 (95.54%) of 27,982 records across the 9 datasets. When targeting exclusion, the weighted average precision and recall reached approximately 1.00 (SD < 0.01; range 0.99-1.00) and 0.96 (SD 0.04; range 0.90-1.00), respectively. In contrast, when targeting inclusion, the weighted average precision was 0.15 (SD 0.13; range 0.05-0.47), while the weighted recall was 0.90 (SD 0.16; range 0.62-1.00). *F*_1_-scores for included articles were modest, with a weighted average of 0.22 (SD 0.16; range 0.04-0.58). Detailed inclusion and exclusion status and more detailed performance metrics are presented in Tables S1 and S2 in Multimedia Appendix 1. Furthermore, the paired *t* test comparing the mean κ (0.258) and the mean PABAK (0.873) revealed a statistically significant difference (*t_8_*=−11.740; *P*<.001), confirming that class imbalance biased κ downward and justifying the use of PABAK for a more accurate assessment of consistency. The weighted mean PABAK value across all datasets was high at 0.91 (range 0.76-0.99), indicating excellent overall consistency of the model predictions. The calculation results of the significance test of the difference between κ and PABAK are provided in Multimedia Appendix 1. In addition, the 3 metrics for imbalanced datasets, F-β, balanced accuracy, and MCC, demonstrated moderate performance levels. When β was set to 2, the weighted average of F-β was 0.45 (range 0.09-0.68). The weighted average of balanced accuracy was 0.93 (range 0.81-0.99). The weighted average of MCC was 0.27 (range 0.14-0.58). More detailed information about imbalance metrics is provided in Table S3 in Multimedia Appendix 1.

**Figure 3:**
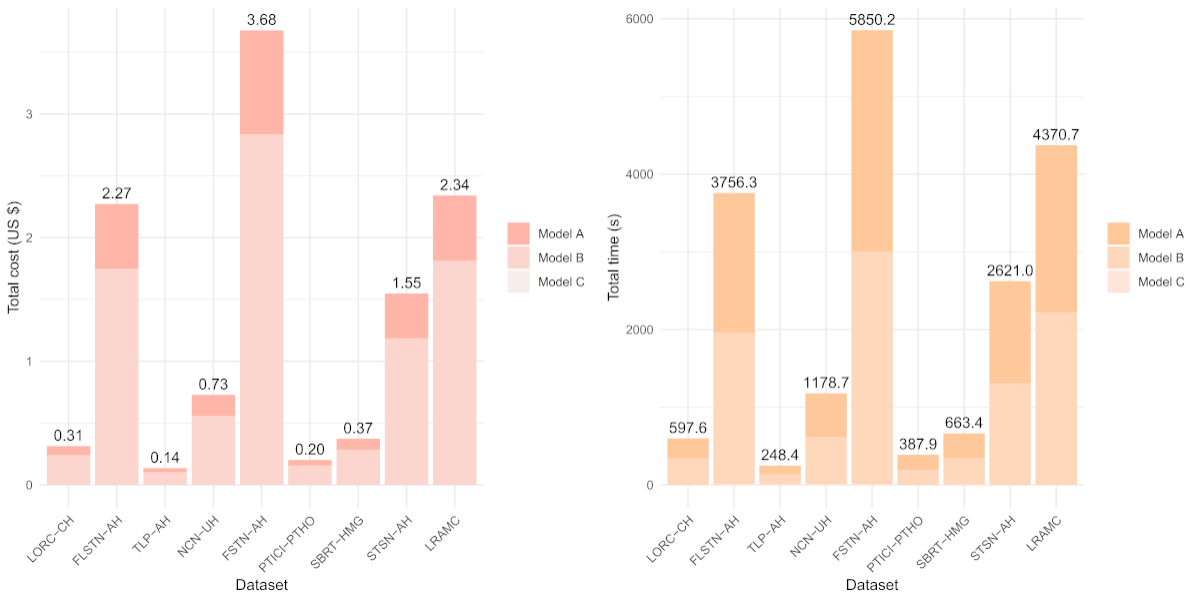
Comparative analysis of financial and time costs for the system across 9 datasets. (A) Financial costs (in US $) associated with analyzing each validation dataset using 3 large language models (LLMs): model A (Doubao-1.5-pro-32k), model B (DeepSeek-v3), and model C (DeepSeek-R1-Distill-Qwen-7B). Each stacked bar represents the total cost per dataset, with segments corresponding to the expenditure for each individual model. (B) Time costs (in seconds) required by the same 3 LLMs to process each dataset. Stacked segments similarly represent the time consumed by each model. This figure provides a direct visual comparison of the distribution and overall scale of economic and temporal resources consumed by each model during the literature screening process across different validation datasets. FLSTN-AH: first-line systemic therapy network meta-analysis for advanced hepatocellular carcinoma; FSTN-AH: frontline systemic therapy network meta-analysis for advanced hepatocellular carcinoma; LORC-CH: laparoscopic versus open resection in cirrhotic hepatocellular carcinoma; LRAMC: liver resection versus ablation within Milan criteria; NCN-UH: novel combination network meta-analysis for unresectable hepatocellular carcinoma; PTICI-PTHO: pre-transplant immune checkpoint inhibitors impact on post-transplant hepatocellular carcinoma outcomes; SBRT-HMG: stereotactic body radiation therapy in hepatocellular carcinoma meta-analysis and guidelines; STSN-AH: systemic treatment sequencing network meta-analysis for advanced hepatocellular carcinoma; TLP-AH: transarterial chemoembolization plus lenvatinib plus programmed death receptor 1 inhibitor in advanced hepatocellular carcinoma.

In terms of time and economic costs, as this system used cloud-based LLM calls and processing, most of the time and monetary costs depended on the processing time and pricing of the cloud computing platform. On the basis of the processing capacity and quotation of Volcano Engine (a cloud computing platform in mainland China), under the default hyperparameter settings, the system’s expenditure could be considered low, as shown in Figure 3. Regarding time costs, 19,674.2 seconds (approximately 5 h and 28 min) were spent analyzing the 9 datasets, with an average of 2186.0 seconds (approximately 36 min; SD 2052.4 seconds) per dataset. In addition, the time required to analyze each 1000 entries was approximately 703.1 seconds (approximately 12 min). Among these datasets, the processing time ranged from 248.4 seconds (approximately 4 min [the shortest] observed for transarterial chemoembolization plus lenvatinib plus programmed death receptor 1 inhibitor in advanced hepatocellular carcinoma [TLP-AH];12/309, 3.9%) to 5850.2 seconds (approximately 1 h and 37 min [the longest] observed for the frontline systemic therapy network meta-analysis for advanced hepatocellular carcinoma [FSTN-AH]; 15/8733, 0.17%). Furthermore, the economic costs were modest, with the analysis of all datasets totaling US $11.59, an average of US $1.29 (SD US $1.25) per dataset, and approximately US $0.41 per 1000 entries. Among the individual datasets, the smallest (TLP-AH) incurred a cost of US $0.14, whereas the largest (FSTN-AH) incurred a cost of US $3.68. These fluctuations in time and economic costs underscore the computational resource implications associated with disparate dataset characteristics, as further elucidated in Tables S4 and S5 in Multimedia Appendix 1.

## Discussion

### Principal Findings

Automation in the literature screening process during meta-analysis can save scholars significant time and effort. Targeting inclusion, our system achieved a weighted average accuracy of 0.96 and a weighted average PABAK value of 0.91. In addition, the system achieved a weighted average recall of 0.90, with evaluations across multiple datasets showing a recall value of 1.00. This suggests that our system and approach may have potential practical use. Furthermore, our system leverages a program that sends local requests to the LLM’s cloud computing service, resulting in minimal requirements for user-side devices. It does not necessitate high-performance GPUs, and only terminal devices capable of multithreading are required, which greatly facilitates user deployment. Through the multithreaded design combined with certain cloud computing services, the processing duration ranged from approximately 4.1 minutes for the quickest run to approximately 1.6 hours for the most extensive one, thereby saving users’ time. Simultaneously, owing to our selection of LLMs that are not mainstream top-tier ones, the costs remain low. On specific platforms, the analysis of our most complex dataset incurred <US $4, substantially expanding practical accessibility for researchers with limited budgets. Regarding scalability considerations, the system’s multithreaded architecture can be enhanced by increasing the number of threads to achieve greater parallelism, thereby reducing processing times for larger datasets. In addition, expanding batch processing sizes would optimize LLM invocations, allowing more efficient handling of high-volume data. Finally, adopting more optimized models with faster inference could further improve performance and adaptability, ensuring viability for extensive real-world meta-analyses while maintaining modest resource demands.

A cornerstone of our system is the collaborative integration of multiple LLMs, drawing inspiration from the tripartite collaborative framework in cognitive science [30]. This design enables decision sharing among language models through the transmission of information, allowing subsequent models to optimize their judgments based on the results of preceding models. Such an iterative approach not only improves analytic accuracy but also enhances interpretability, partially mirroring the collaborative nature of manual meta-analysis. A previous study [19] has used a single GPT-4o model for similar classification tasks, achieving an accuracy of 0.91, whereas our system achieved a weighted average accuracy of 0.96, outperforming this prior work. Unlike prior studies reliant on a single model, such as ChatGPT, our system uses 3 distinct LLMs, which offer performance comparable to GPT-4o at reduced costs, and an advanced open-source reasoning model that further lowers deployment expenses [19,20,31].

Presently, there are already several tools that use AI in a comparable manner. These include Scispace, Elicit, Consensus, and Scite, which use literature titles and abstract information to leverage LLMs to extract key insights from literature and integrate them. However, most of these platforms are primarily designed for conducting literature reviews, and these supporting systematic review analyses often come with high economic costs and lack transparency. In addition, platforms such as DistillerSR and RobotAnalyst have automated the systematic review process [32,33]. However, these tools are not currently strongly integrated with LLMs. In contrast, our system combines the strengths of LLMs and programmatic pipelines within its workflow to support the updating of systematic reviews and meta-analyses.

Our system has certain limitations. Although our datasets encompass multiple topics with substantial numbers of records initially identified, the Sankey diagram, as shown in Table S6 and Figure S1 in Multimedia Appendix 1, revealed that data flow across datasets exhibits remarkable consistency, indicating limited divergent perspectives generated by the secondary model. Refinements to model temperature and prompt engineering may prove beneficial in addressing this limitation [34,35]. Crucially, it should not be ignored that our overall precision and *F*_1_-scores are relatively low, as shown in Figure 2. We conducted an in-depth analysis of the cases where misidentification occurred. First, nonspecialized models and potentially insufficient training corpora may be responsible for the initial misclassification of literature entries, as they fail to accurately grasp the key conceptual features within the texts, leading to incorrect categorization. For example, we found that cost-effectiveness analyses derived from RCTs published by other researchers were incorrectly identified as RCTs themselves. Providing a detailed description of key concepts in the prompt or collaboratively using a chain of knowledge approach may be a potential solution to improve this aspect [36]. In addition, the degradation in contextual understanding under long prompt contexts may limit model performance, which could be attributed to attention dilution [37]. Reducing batch size may mitigate this issue. Our selected model, DeepSeek-V3, being a distilled version, exhibits limitations in conceptual transformation and performance [38]. This phenomenon can be ascribed, at least in part, to the model collapse problem inherent to LLM [39,40]. Model collapse is a systematic degradation phenomenon that occurs in generative models during recursive training, which manifests itself as loss of output diversity, reduced semantic coherence, and even logical confusion [40]. This problem can be optimized in subsequent training of LLMs, such as through the use of data specific to the treatment of liver cancer to train or fine-tune, thereby reducing the risk of overgeneralization or improvements at the architectural level [41,42]. Moreover, our dataset is currently focused on HCC treatment. Although we selected HCC treatment as a complex test case to rigorously evaluate our system, and the workflow is theoretically generalizable, its performance on broader oncology topics or other disease domains remains to be validated.

**Figure 4:**
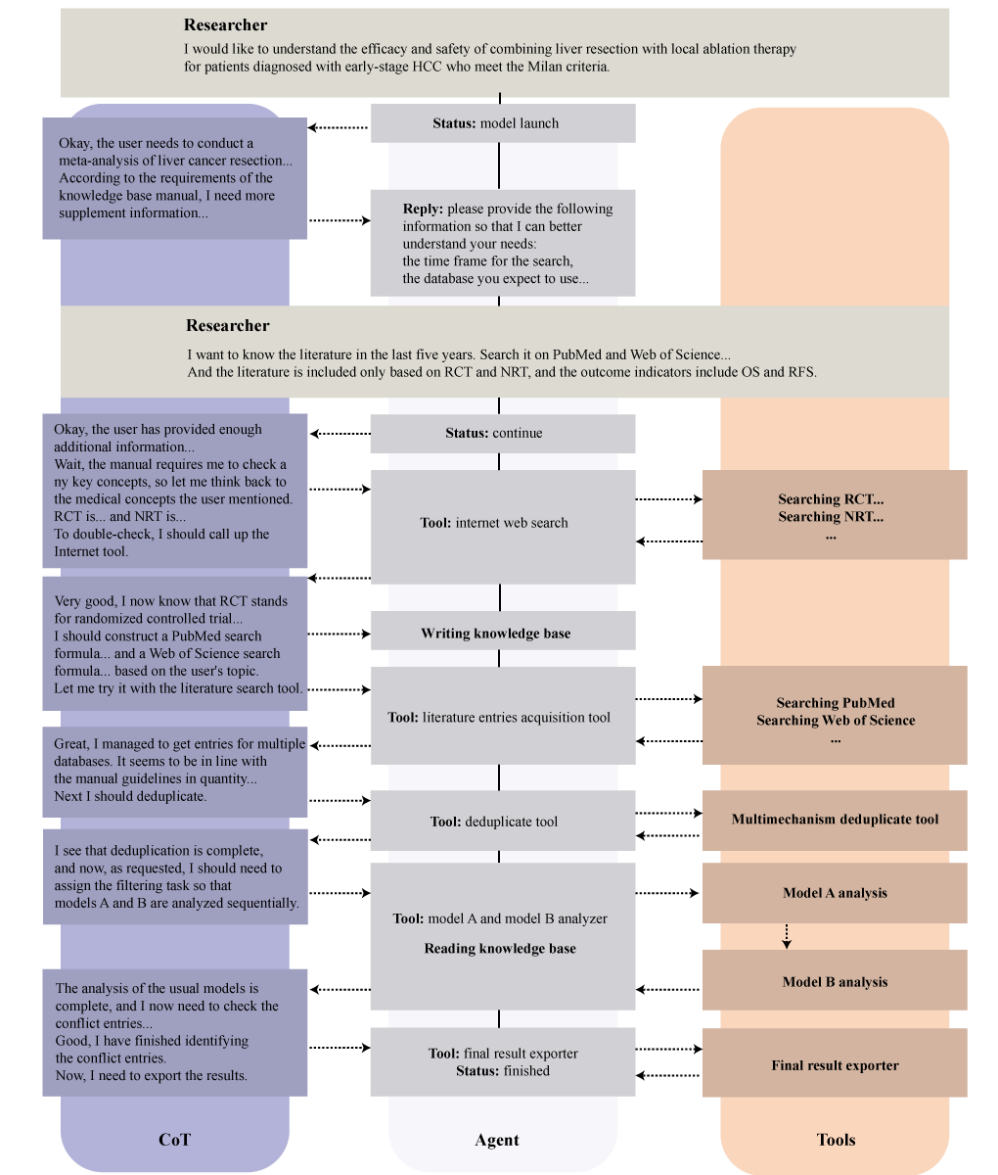
Integrated framework for systematic literature screening using a large language model (LLM) agent and reinforcement learning (RL) optimization. This diagram illustrates the workflow of a systematic literature screening process driven by an integrated LLM agent. The central agent uses a chain of thought (CoT) approach as its core reasoning mechanism, supported by automated tools and optimized throughout by RL. The process starts when the researcher initiates a request, prompting the agent to clarify the analysis scope, including publication timeframe, selected databases, and key outcome indicators. Following this, the central agent uses internet-based searches to verify essential medical concepts. Subsequently, automated tools conduct structured literature searches across databases, retrieving and deduplicating entries for a refined set. The central agent directs models for sequential initial and secondary screenings, then synthesizes results, resolves conflicts, and generates an optimized final output. HCC: hepatocellular carcinoma; RCT: randomized controlled trial; NRT: nonrandomized trial.

Architecture construction based on LLM agents and reinforcement learning (RL) may be a more practical approach to address potential issues, such as the low recall observed in this study. Despite the lack of a unified definition, the LLM agent is driven by a core LLM that acts as the brain, completing the logical chain through collaboration [43-46]. As illustrated in Figure 4, our proposed system uses a reasoning model as the core foundation, leveraging its complete chain of thought capabilities. For ambiguous tasks, such as analyzing the “liver resection versus ablation within Milan criteria” dataset’s objectives (comparing efficacy and safety of liver resection combined with local ablation in early-stage HCC meeting Milan criteria), the model accepts the request, references the knowledge base for guidelines, and queries the researcher on parameters such as publication time range and search platforms. Upon receiving sufficient input, the core model initiates planning: it invokes internet tools to iteratively search key concepts (eg, Milan criteria metrics and local ablation therapies) until a reward threshold is met, storing results in the knowledge base. It then constructs platform-specific search queries, directing a literature acquisition tool to retrieve entries (including titles and abstracts) until the threshold is reached. Subsequently, a deduplication tool processes multiplatform entries to yield unique results. The core model then assigns 2 distinct regular models for primary and secondary screening, referencing the knowledge base, and resolves conflicts to finalize outcomes. This integration of RL and model specialization creates an advanced system, akin to OpenAI’s deep research feature, which eases the step of reviews. Nevertheless, it is important to note that hallucinations in LLMs are difficult to avoid, and appropriate human intervention remains crucial [47]. Key challenges include automating document retrieval across platforms and embedding RL throughout, warranting further empirical validation.

### Conclusions

The results of this study showed that an automated screening process for systematic reviews and meta-analyses based on LLM was feasible in the context of treatment interventions for HCC. Our findings demonstrated the feasibility and effectiveness of an automated literature screening approach leveraging LLMs for systematic reviews and meta-analyses related to treatment interventions in HCC. The proposed methodology exhibited high recall rates and promising accuracy metrics, highlighting its capability to reliably identify relevant literature while reducing both the time and financial resources required for manual screening. The tripartite collaborative integration of general-purpose LLMs with a reasoning-optimized model further enhanced transparency and interpretability throughout the analysis process. We propose refining the current framework by integrating an agent-based workflow augmented with RL, which could further enhance analytic precision and clinical relevance. Subsequent studies are warranted to empirically validate the effectiveness and feasibility of these proposed enhancements.

## Appendix

Multimedia Appendix 1 Supplementary information.

## Abbreviations

AI: artificial intelligence
HCC: hepatocellular carcinoma
LLM: large language model
MCC: Matthews correlation coefficient
PABAK: prevalence-adjusted bias-adjusted κ
PICOS: population, intervention, comparison, outcome, and study design
PLC: primary liver cancer
PRISMA: Preferred Reporting Items for Systematic Reviews and Meta-Analyses
RCT: randomized controlled trial
RL: reinforcement learning

## Data Availability

The datasets generated or analyzed during this study are available from the corresponding author on reasonable request. A free demo is hosted on Hugging Face [48]. The source code is openly available on GitHub [49].
