# Supplementary material for: Automated Literature Screening for Hepatocellular Carcinoma Treatment Through Integration of 3 Large Language Models: Methodological Study
Source: JMIR Med Inform. 2025 Sep 8;13:e76252. doi: 10.2196/76252 (PMC12455167; doi:10.2196/76252)
Supplement: Multimedia Appendix 1 [file medinform_v13i1e76252_app1.docx]

Supplementary Materials

Supplementary Material of Automated Literature Screening for Hepatocellular Carcinoma Treatment

Contents

[1 Supplementary Materials 4](#_Toc23349)

[1.1 A1 Deduplication design 4](#_Toc27409)

[1.2 A2 Prompts for each model 7](#_Toc32176)

[1.3 A3 Hyperparameters for each model 13](#_Toc20911)

[1.4 A4 Methodology for analyzing process construction 14](#_Toc31442)

[1.5 A5 Formulas for calculating the statistical metrics 17](#_Toc30197)

[1.6 A6 Design of the simulation program for economic cost estimation 19](#_Toc12052)

[1.7 A7 PICOS of each dataset 21](#_Toc6546)

[1.8 A8 Search terms used for constructing each dataset 27](#_Toc6197)

[1.9 A9 Comparative test results of kappa and PABAK 37](#_Toc8937)

[2 Supplementary Tables 38](#_Toc27684)

[Table S1 The screening status of the number of literature from various datasets by the analysis system 38](#_Toc7390)

[Table S2 The performance of the analysis system across various datasets in the exclusion and inclusion target 39](#_Toc19488)

[Table S3 Performance of imbalanced statistical metrics across nine datasets 40](#_Toc1525)

[Table S4 Platform unit prices and estimated analysis costs for each model 41](#_Toc7643)

[Table S5 Detailed time and financial costs of the three large language model-based analytical system across nine datasets 42](#_Toc29035)

[Table S6 Reversion data during the analysis of Model B 43](#_Toc8866)

[3 Supplementary Figures 44](#_Toc21054)

[Figure S1 Information flow of LLMs in literature screening on nine datasets. 44](#_Toc25814)

# Supplementary Materials

## A1 Deduplication design

Given the significant engineering effort required to implement automatic correction of bibliographic metadata via API calls, and considering that exporting deduplicated records through existing reference management tools often results in the loss of abstract information, we developed a lightweight deduplication program to merge duplicate bibliographic entries. The program follows a three-stage, hierarchical approach.

First, bibliographic entries, each containing a *title*, *author*, and *abstract*, were extracted from the DataFrame and indexed using a source_mapping dictionary that records the original database source and index (in the format "*database_name:original_index*"). Next, a preprocessing function was applied to normalize the text: special characters were removed, all text was converted to lowercase, and common stop words were filtered out.

In the first stage, entries sharing identical DOIs were merged. Entries not matched in this stage proceeded to the second stage, where similarity matching was performed based on both title and author information. A clustering algorithm grouped entries whose similarity exceeded a predefined threshold. In the final stage, remaining unmatched entries were clustered based on the combined similarity of titles and abstracts, followed by merging within clusters.

For both the second and third stages, cosine similarity was computed using Scikit-learn’s TfidfVectorizer on the relevant text fields. TF-IDF is a weight indicator composed of two parts:

$$TF-IDF(t,d)=\mathrm{TF}(t,d)\times IDF(t)$$

Where $\text{t}$: represents a term in the corpus. $\text{d}$: represents a document within the corpus. $\text{TF(}\text{t}\text{,}\text{d}\text{)}$: denotes the Term Frequency, which measures the frequency of term $\text{t}$ appearing in document $\text{d}$. $\text{IDF(}\text{t}\text{)}$: denotes the Inverse Document Frequency, which quantifies the rarity of term $\text{t}$ across the entire corpus. The Term Frequency (TF) was calculated as:

$$\mathrm{TF}(t,d)=\frac{f_{t,d}}{\sum_{k} f_{k,d}}$$

Where $k$ is the all terms in document $d$. The Inverse Document Frequency (IDF) was computed as:

$$\mathrm{IDF}(t)=\log\left( \frac{N+1}{n_{t}+1} \right)+1$$

Where $\text{N}$: represents the total number of documents in the corpus. $\text{n}_{\text{t}}$: represents the number of documents in the corpus that contain term $\text{t}$. Using these metrics, TfidfVectorizer transformed the textual data into a sparse term-document matrix, where each dimension corresponds to a unique term and each value reflects the term’s weighted importance. Cosine similarity between entries was then calculated as:

$$cosine\_sim[i,j]=\frac{\sum_{k=1}^{m} a_{ik}\cdot a_{jk}}{\sqrt{\sum_{k=1}^{m} a_{ik}^{2}}\cdot\sqrt{\sum_{k=1}^{m} a_{jk}^{2}}}$$

Where $\text{a}_{\text{iK}}$: represents the TF-IDF weight of the $\text{k}$-th term in the $\text{i}$-th document. $\text{a}_{\text{jk}}$: represents the TF-IDF weight of the $\text{k}$-th term in the $\text{j}$-th document. $\text{m}$: represents the total number of unique terms in the vocabulary of the corpus. Following vectorization, a Union-Find (Disjoint Set) clustering algorithm was employed to group similar entries. The procedure was implemented as follows: 1) A parent array was initialized, with each entry initially pointing to itself as its root; 2) The find function recursively located the root of a given node (with path compression for efficiency), and the union function merged two sets by linking their roots; 3) Any pair of entries with a cosine similarity above a threshold (set to 0.8 by default) were merged into the same cluster. Through this hierarchical process, duplicate and near-duplicate bibliographic entries were effectively consolidated into distinct clusters, yielding a final deduplicated dataset.

## A2 Prompts for each model

**Model A**

You are a medical research expert analyzing clinical trial abstracts.

Your task is to analyze each article and determine if it matches the PICOS criteria.

Target PICOS criteria:

- Population: {population}

- Intervention: {intervention}

- Comparison: {comparison}

- Outcome: {outcome}

- Study Design: {study_design}

Input articles:

{abstracts_json}

Each article in the input contains:

- index: article identifier

- title: article title

- abstract: the text to analyze

IMPORTANT: You must:

1. Read both the title and abstract carefully

2. Consider information from both title and abstract in your analysis

3. Pay special attention to any discrepancies between title and abstract

4. Follow these strict JSON formatting rules:

   - Use double quotes for all strings

   - Ensure all strings are properly terminated

   - Use commas between array items and object properties

   - Do not use trailing commas

   - Keep the response concise and avoid unnecessary whitespace

   - Escape any special characters in strings

   - Use true/false (not True/False) for boolean values

5. CRITICAL: Every field in the response MUST contain a value:

   - NEVER leave any field empty or use null values

   - If information is not found, use "not specified" or "not found in text"

   - For decisions, always provide either true or false

   - For reasons, always provide a clear explanation

Provide your analysis in this exact JSON format:

{{

  "results": [

    {{

      "Index": "ARTICLE_INDEX",

      "A_P": "brief population description",

      "A_I": "brief intervention description",

      "A_C": "brief comparison description",

      "A_O": "brief outcome description",

      "A_S": "brief study design description",

      "A_Decision": true/false,

      "A_Reason": "brief reasoning for match/mismatch"

    }},

    ...

  ]

}}

Keep all descriptions brief and focused. Do not include line breaks or special characters in the text fields.

If any field is not found in both title and abstract, use "not specified" as the value.

Be strict in your evaluation and ensure the output is valid JSON format.

**Model B**

You are a critical reviewer in a systematic review team.

Your task is to rigorously scrutinize Model A's analysis and provide your own assessment.

You should actively look for potential flaws or oversights in Model A's analysis, while maintaining a high standard of evidence-based evaluation.

Target PICOS criteria:

- Population: {population}

- Intervention: {intervention}

- Comparison: {comparison}

- Outcome: {outcome}

- Study Design: {study_design}

Input articles:

{abstracts_json}

Each article in the input contains:

- Index: article identifier

- title: article title

- abstract: original article abstract

- model_a_analysis:

  - A_P: Model A's population description

  - A_I: Model A's intervention description

  - A_C: Model A's comparison description

  - A_O: Model A's outcome description

  - A_S: Model A's study design description

  - A_Decision: Model A's inclusion decision

  - A_Reason: Model A's explanation

Your task is to:

1. Thoroughly examine both the title and abstract

2. Critically review Model A's PICOS extraction, actively seeking potential issues:

   - Look for missing details or nuances in population characteristics

   - Check for precise intervention specifications

   - Verify completeness of comparison group description

   - Examine outcome measurements and their relevance

   - Scrutinize study design classification

3. Consider any discrepancies between title and abstract information

4. Provide corrections with evidence from both title and abstract:

   - B_P: Your corrected population description (use "-" only if A_P is completely accurate)

   - B_I: Your corrected intervention description (use "-" only if A_I is completely accurate)

   - B_C: Your corrected comparison description (use "-" only if A_C is completely accurate)

   - B_O: Your corrected outcome description (use "-" only if A_O is completely accurate)

   - B_S: Your corrected study design description (use "-" only if A_S is completely accurate)

5. Make your own independent inclusion decision (B_Decision)

6. Provide detailed reasoning (B_Reason) that:

   - Points out any oversights or inaccuracies in Model A's analysis

   - Cites specific evidence from both title and abstract

   - Explains why your corrections or agreements are justified

IMPORTANT: You must follow these strict JSON formatting rules:

1. Use double quotes for all strings

2. Ensure all strings are properly terminated

3. Use commas between array items and object properties

4. Do not use trailing commas

5. Keep the response concise and avoid unnecessary whitespace

6. Escape any special characters in strings

7. Use true/false for B_Decision (true means the article should be included)

8. CRITICAL: Every field in the response MUST contain a value:

   - NEVER leave any field empty or use null values

   - For B_P, B_I, B_C, B_O, B_S: use "-" if you agree with Model A, otherwise provide correction

   - For B_Decision: always provide either true or false

   - For B_Reason: always provide a detailed explanation

Return your analysis in this exact JSON format:

{{

  "results": [

    {{

      "Index": "ARTICLE_INDEX",

      "B_Decision": true/false,

      "B_Reason": "detailed reasoning with evidence",

      "B_P": "-" or "corrected population description with evidence",

      "B_I": "-" or "corrected intervention description with evidence",

      "B_C": "-" or "corrected comparison description with evidence",

      "B_O": "-" or "corrected outcome description with evidence",

      "B_S": "-" or "corrected study design description with evidence"

    }},

    ...

  ]

}}

Keep descriptions focused and evidence-based. Do not include line breaks or special characters.

Use "-" only when you are completely certain that Model A's extraction is accurate and complete.

Your B_Decision should be based on whether the article meets all PICOS criteria.

Remember to be thorough in your critique while maintaining objectivity and evidence-based reasoning.

**Model C**

You are the final arbitrator in a systematic review team.

Your task is to analyze the assessments from Model A and Model B, and make a final decision.

Target PICOS criteria:

- Population: {population}

- Intervention: {intervention}

- Comparison: {comparison}

- Outcome: {outcome}

- Study Design: {study_design}

Input articles:

{abstracts_json}

Each article in the input contains:

- Index: article identifier

- title: article title

- abstract: original article abstract

- model_a_analysis: Model A's assessment

- model_b_analysis: Model B's assessment

Your task is to:

1. Review both the title and abstract thoroughly

2. Compare Model A and Model B's assessments

3. Consider any discrepancies between title and abstract information

4. Make a final decision considering:

   - Accuracy of PICOS criteria matching

   - Validity of reasoning from both models

   - Evidence from both title and abstract

5. Provide your final assessment:

   - C_Decision: final inclusion decision

   - C_Reason: detailed explanation of your decision

   - Note any disagreements between models and how you resolved them

IMPORTANT: You must follow these strict JSON formatting rules:

1. Use double quotes for all strings

2. Ensure all strings are properly terminated

3. Use commas between array items and object properties

4. Do not use trailing commas

5. Keep the response concise and avoid unnecessary whitespace

6. Escape any special characters in strings

7. CRITICAL: Every field in the response MUST contain a value:

   - NEVER leave any field empty or use null values

   - For C_Decision: always provide either true or false

   - For C_Reason: always provide a detailed explanation of your decision

Return your analysis in this exact JSON format:

{{

  "results": [

    {{

      "Index": "ARTICLE_INDEX",

      "C_Decision": true/false,

      "C_Reason": "detailed reasoning with evidence"

    }},

    ...

  ]

}}

Keep your reasoning focused and evidence-based.

Your C_Decision should be based on whether the article truly meets all PICOS criteria.

Be thorough in your analysis while maintaining objectivity.

## A3 Hyperparameters for each model

For the three large language model requests, the same hyperparameters were used, except that Model C enable the reasoning model opinion for parsing. The common configurations were as follows: (1) temperature of 0.3; (2) batch size of 10; (3) maximum tokens of 4096; (4) threads of 8, and (5) timeout of 180 s. The remaining hyperparameters were kept to the platform's default values.

## A4 Methodology for analyzing process construction

The development of an analysis system based on three large language models was motivated by two real-world scenarios. The first scenario involves the screening process in meta-analyses, which is notably intricate and requires substantial time. A considerable proportion of the abstracts identified through manual review are ultimately deemed irrelevant for inclusion. The second scenario concerns the challenge of validating the efficacy of an engineering approach for addressing errors, which is inherently complex.

Based on objective requirements, including a survey of previous research, it was found that large language models can potentially replace human effort in literature screening or, at a minimum, conduct preliminary screening. However, there is currently a paucity of examples demonstrating the use of large language models in the screening process of systematic reviews and meta-analyses. Most existing tools focus on summarizing literature reviews. This apparent gap may be attributed to copyright constraints related to database entries, coupled with the prevalence of knowledge barriers in many databases.

It is imperative to employ an appropriate programming language for process design. In our effort to optimize the relevance of LLM back-end requests and minimize development time, we used Python 3.12.3 and managed our development environment with Conda. Nonetheless, we refrained from using OpenAI’s Python package and instead implemented our own HTTP POST method, primarily to enable asynchronous multithreading and high concurrency, thereby minimizing analysis time as much as possible.

The first module of the entire analysis workflow—the parsing of literature citation files—is executed via a custom-developed program. This program reads each line in a streaming manner and processes the content of each field as it is retrieved. We rely on NBIB files from PubMed, as well as RIS files from databases such as Web of Science, which are structured as follows:

TI – Treatment of Liver Cancer.
LID – 11.0000/00 [doi]

Although the data structure is amenable to parsing, it does not offer the convenience of JSON. Fields and field combinations (e.g., title, or title and author) in the exported tabular file are converted into term frequency–inverse document frequency (TF-IDF) vectors using the *TfidfVectorizer* class from the *scikit-learn* package. This procedure ensures that each entry is transformed into a unique vector representation. Subsequently, entries with similar features are clustered, and only one entry is retained.

The deduplication process has encountered challenges, including repeated abstracts and titles, sometimes with different authors. To address these issues, a refined approach has been developed to reduce duplicate entries without excluding too many valid ones. Nonetheless, some potential problems may remain undiscovered.

Model information flow is achieved through JSON appending. Initially, Model A inserts the target PICOS and the deduplicated title and abstract fields of a batch of document entries (in our experiment, 10 entries per batch) into a predefined prompt via JSON serialization. The data is then transmitted to the back end using the multithreaded batch process we designed. Once Model A has processed all entries in the dataset, the results are stored. Subsequently, Model B begins its operation. Model B also reads the target PICOS and batch-reads the literature entries but additionally considers Model A’s data—its decisions and justifications—thereby using the output of Model A as part of its own input.

When Model B completes its analysis, Model C is triggered. It checks any literature entries on which Models A and B disagree and refers to their decisions and justifications, as well as the original abstracts and titles, to make a final determination. For Model C, an inference model based on reinforcement learning is employed; thus, the theoretical time cost is higher. However, in practice, this design was rarely invoked in the tested datasets. The data returned by all models upon completion is in JSON format, facilitating its deserialization into tabular data.

## A5 Formulas for calculating the statistical metrics

The statistical metrics used in our study include accuracy, precision, recall, F1-score, kappa, and prevalence-adjusted bias-adjusted kappa (PABAK). The formula for accuracy is:

$$Accuracy=\frac{TP+TN}{TP+TN+FP+FN}$$

Where $\text{TP}$ (True Positives) refers to the number of relevant studies correctly identified by the system as "included"; $\text{TN}$ **(True Negatives)** refers to the number of irrelevant studies correctly identified as "excluded"; $\text{FP}$ **(False Positives)** refers to the number of irrelevant studies incorrectly classified as "included"; $\text{FN}$ **(False Negatives)** refers to the number of relevant studies incorrectly classified as "excluded". The formulas for precision are:

$$Precision_{inclusion}=\frac{TP}{TP+FP}$$

$${Precision}_{exclusion}=\frac{TN}{TN+FN}$$

Furthermore, the formulas for recall are:

$$Recall_{inclusion}=\frac{TP}{TP+FN}$$

$${Recall}_{exclusion}=\frac{TN}{TN+FP}$$

The F1 score emphasizes the incorporation of both precision and recall, and its formula is:

$$F1=\frac{2\cdot{Precision}_{inclusion}\cdot{Recall}_{inclusion}}{{Precision}_{inclusion}+{Recall}_{inclusion}}$$

The kappa value, also known as Cohen’s Kappa, is an important statistical metric for measuring the agreement and reliability of classifiers, and its formula is:

$$\kappa=\frac{P_{o}-P_{e}}{1-P_{e}}$$

Where $P_{o}$ is observed agreement, and its formula is:

$$P_{o}=\frac{TP+TN}{TP+TN+FP+FN}$$

$P_{e}$ is expected agreement by chance, and its formula is:

$$P_{e}=\frac{(TP+FP)(TP+FN)+(FN+TN)(FP+TN)}{(TP+TN+FP+FN)^{2}}$$

The prevalence-adjusted bias-adjusted kappa (PABAK) is a variant of kappa that corrects for prevalence and bias, especially useful for imbalanced datasets. Its formula is:

$$PABAK=2P_{o}-1$$

Where $\text{P}_{\text{o}}$ is the observed agreement as defined above.

## A6 Design of the simulation program for economic cost estimation

The program implements a cost analysis framework for evaluating the computational expenses of three large language models, referred to as Models A, B, and C, in the context of a systematic review process for screening clinical trial abstracts based on predefined Population, Intervention, Comparison, Outcome, and Study Design (PICOS) criteria. The cost estimation is performed by simulating LLM interactions in batches, leveraging pre-computed outputs stored in an excel file, and calculating token-based expenditures using predefined per-model pricing rates (in USD per 1,000 tokens). Specifically, each model has distinct input and output rates defined in the code.

The analysis proceeds in three sequential phases, with costs accumulated across all processed entries (filtered to exclude empty abstracts, resulting in a dataset synchronized between input data from data.xlsx and results). In the first two phases, the dataset is divided into batches. For each batch, prompts are constructed by formatting predefined templates with PICOS details and serialized JSON representations of article metadata (including indices, titles, and abstracts). Input tokens are counted for these prompts using the tiktoken library (based on the GPT-3.5-Turbo encoding). Outputs are derived directly from the pre-stored results in the final Excel file, serialized to JSON, and similarly tokenized. Costs for each batch are computed as the sum of (input tokens / 1,000 × input rate) and (output tokens / 1,000 × output rate), then aggregated across batches to yield total costs and token counts for the respective models.

The third phase focuses exclusively on conflicting entries, those where the first two models disagree on inclusion decisions, identified during the earlier phases. These conflicts are batched, with analogous prompt construction incorporating both prior model analyses. Token counting and cost calculation mirror the earlier phases, using pre-stored outputs from the results file. Finally, aggregate metrics (total costs, input/output tokens, and overall tokens per model) are compiled, including a grand total, and exported to a CSV file for tabular reporting. This approach ensures a granular, phase-wise breakdown of costs while accounting for real-world LLM usage patterns in multi-stage decision-making workflows.

## A7 PICOS of each dataset

**LORC-CH**

"population": "Patients with hepatocellular carcinoma (HCC) in the setting of liver cirrhosis.",

"intervention": "Laparoscopic liver resection (LLR).",

"comparison": "Open liver resection (OLR).",

"outcome": "Overall survival (OS) or Intraoperative outcomes (operation duration, blood loss, blood transfusion, Pringle manoeuvre utilization), postoperative outcomes (overall complications, major complications, length of hospital stay, 90-day mortality) and oncological outcomes (R0 resection rates).",

"study_design": "Randomised controlled trials (RCTs) or propensity score-matched (PSM) studies."

**FLSTN-AH**

"population": "Adults suffer from unresectable or advanced hepatocellular carcinoma",

"intervention": "Various preferred systemic treatment options, including immune checkpoint inhibitors combined with anti-angiogenic therapy (e.g., anti-PD-(L)1/VEGF antibody combinations) and other novel combinations (e.g., tremelimumab + durvalumab), as well as single-agent targeted therapies (e.g., sorafenib, lenvatinib, etc.).",

"comparison": "The primary control are either conventional standard treatment (e.g., sorafenib) or placebo control; different comparison groups may have been used in each RCT to ensure that the efficacy of each treatment regimen could be compared indirectly through network comparisons.",

"outcome": "Overall Survival (OS) or Progression-Free Survival (PFS) or safety (including incidence of serious adverse events).",

"study_design": "The study selection are strictly limited to randomized controlled trials (RCTs) as the primary research methodology. This exclusion criteria extended to all other study designs including meta-analyses, case reports, case series, observational studies, and qualitative research. Furthermore, any form of effect size analysis, efficacy evaluation, or outcome measurement interpretation was deliberately omitted from the research scope to maintain focus on trial methodology and design characteristics rather than therapeutic outcomes."

**TLP-AH**

"population": "Patients with advanced hepatocellular carcinoma (HCC) who are unsuitable for curative treatments (e.g. surgical resection or liver transplantation), including cases with portal vein tumor thrombus or extrahepatic metastasis.",

"intervention": "Transarterial chemoembolization (TACE) combined with lenvatinib and a PD-1 inhibitor.",

"comparison": "Other treatment regimens, including but not limited to: TACE alone, TACE combined with sorafenib, TACE combined with lenvatinib, Lenvatinib plus PD-1 inhibitor, TACE plus sorafenib with PD-1 inhibitor",

"outcome": "Overall survival (OS) or progression-free survival (PFS) or objective response rate (ORR) or disease control rate (DCR) or incidence and severity of adverse events (AEs).",

"study_design": "This study specifically targets observational cohort studies, case-control studies, and randomized controlled trials (RCTs) with quantifiable outcome data, while excluding meta-analyses, systematic reviews, case reports/series, editorials, and investigations focused on benefit quantification or analogous methodologies."

**NCN-UH**

"population": "Patients with unresectable hepatocellular carcinoma (uHCC) who are treatment‐naïve for systemic therapy.",

"intervention": "First-line systemic therapies including immune-checkpoint inhibitors (alone or in combination, e.g. PD-1/PD-L1 inhibitors, CTLA-4 inhibitors, or their combinations with anti-VEGF agents) and tyrosine kinase inhibitors (such as sorafenib, lenvatinib, and donafenib).",

"comparison": "Comparator arms are either placebo or the standard treatment (sorafenib) as used in the respective phase III trials.",

"outcome": "Overall survival (OS) or progression-free survival (PFS) or objective response rate (ORR) or safety parameters (incidence of adverse events or treatment discontinuations).",

"study_design": "Only Phase III randomized controlled trials (RCTs) were considered for inclusion, with explicit exclusion of secondary analyses including cost-benefit analyses, meta-analyses, systematic reviews, case reports, and all other non-original research designs."

**FSTN-AH**

"population": "Patients with unresectable hepatocellular carcinoma (uHCC) who are treatment-naive for systemic therapy.",

"intervention": "First-line systemic therapies including immune-checkpoint inhibitors (alone or in combination, e.g. PD-1/PD-L1 inhibitors, CTLA-4 inhibitors, or their combinations with anti-VEGF agents) and tyrosine kinase inhibitors (such as sorafenib, lenvatinib, and donafenib).",

"comparison": "Comparator arms are either placebo or the standard treatment (sorafenib) as used in the respective phase III trials.",

"outcome": "Primary outcomes included post-transplant allograft rejection, hepatocellular carcinoma recurrence, and overall survival.",

"study_design": "Studies providing individual patient data (IPD) from case reports, case series, and retrospective cohorts were included, while meta-analyses, effect analyses, and studies without IPD (or with non-HCC cases or ICI use only after LT) were excluded."

**PTICI-PTHO**

"population": "Patients diagnosed with hepatocellular carcinoma (HCC) who underwent liver transplantation after receiving pre‐transplant immune checkpoint inhibitor (ICI) therapy.",

"intervention": "Administration of immune checkpoint inhibitors—including anti-PD-1, anti-PD-L1, and CTLA-4 agents—given prior to liver transplantation as a bridging or downstaging strategy.",

"comparison": "Due to the predominance of single-arm case reports/series, a formal comparator was not required.",

"outcome": "Primary outcomes included post-transplant allograft rejection, hepatocellular carcinoma recurrence, and overall survival.",

"study_design": "Studies providing individual patient data (IPD) from case reports, case series, and retrospective cohorts were included, while meta-analyses, effect analyses, and studies without IPD (or with non-HCC cases or ICI use only after LT) were excluded."

**SBRT-HMG**

"population": "Patients with liver-confined hepatocellular carcinoma (HCC).",

"intervention": "Treatment with stereotactic body radiation therapy (SBRT) and SBRT was delivered as a definitive treatment.",

"comparison": "There was no specific comparator required; the focus was on assessing outcomes following SBRT.",

"outcome": "Reporting of long-term outcomes like local control (LC) or overall survival (OS) rates or along with data on hepatic toxicity.",

"study_design": "Retrospective or prospective observational studies with larger than 10 patients only, excluding reviews, meta-analyses, effect analyses, and case reports."

**STSN-AH**

"population": "Adult patients diagnosed with advanced hepatocellular carcinoma (HCC) – that is, patients with unresectable or metastatic liver cancer, generally with ECOG performance status <2 and Child–Pugh A liver function.",

"intervention": "Systemic therapies including: VEGF inhibitors (tyrosine kinase inhibitors [TKIs] and monoclonal antibodies), Checkpoint inhibitors (CPIs), Combinations thereof (e.g., atezolizumab plus bevacizumab) and so on.",

"comparison": "Comparative agents within systemic therapy regimens – namely: Other VEGF inhibitors or CPIs (e.g., sorafenib, lenvatinib, nivolumab) or Placebo (in refractory/second-line settings).",

"outcome": "Primary outcomes were overall survival (OS) and progression-free survival (PFS).",

"study_design": "Inclusion limited to phase III randomized controlled trials (RCTs) published in English. Excluded are meta-analyses, effect analyses, case reports, and other non–original clinical trial designs."

**LRAMC**

"population": "Any patients diagnosed with hepatocellular carcinoma (HCC) that potentially meet the Milan criteria, e.g. patients with a solitary tumor no more than 5cm in diameter or up to three nodules each no more than 3cm, with well‐preserved liver function.",

"intervention": "Liver resection (LR) as the primary curative treatment modality.",

"comparison": "Local ablation therapies including radiofrequency ablation (RFA), microwave ablation (MWA), and RFA combined with trans-arterial chemoembolization (TACE).",

"outcome": "Primary outcome like Overall survival (OS) or Secondary outcomes like Recurrence-free survival (RFS), recurrence pattern (local vs remote recurrence) and overall complications.",

"study_design": "Included Randomized controlled trials (RCTs) and matched non-randomized trials (NRTs) while Meta-analyses, effect analyses, case reports, review articles, and poster presentations are excluded."

## A8 Search terms used for constructing each dataset

**LORC-CH**

*Pubmed*

(("laparoscopic" OR "laparoscopy" OR "minimally invasive") AND ("liver resection" OR "hepatectomy" OR "liver surgery") AND ("hepatocellular carcinoma" OR "HCC" OR "primary liver cancer") AND ("cirrhosis" OR "liver cirrhosis" OR "chronic liver disease")) AND ("1900/01/01"[Date - Publication] : "2020/04/30"[Date - Publication])

*Web of Science*

TS = (( "laparoscopic" OR "laparoscopy" OR "minimally invasive" ) AND ( "liver resection" OR "hepatectomy" OR "liver surgery" ) AND ( "hepatocellular carcinoma" OR "HCC" OR "primary liver cancer" ) AND ( "cirrhosis" OR "liver cirrhosis" OR "chronic liver disease" )) AND PY = 1900-2020 AND DT = (Article OR Abstract)

*Embase*

('laparoscopic' OR 'laparoscopy' OR 'minimally invasive') AND ('liver resection' OR 'hepatectomy' OR 'liver surgery') AND ('hepatocellular carcinoma' OR 'hcc' OR 'primary liver cancer') AND ('cirrhosis' OR 'liver cirrhosis' OR 'chronic liver disease') AND [1900-2020]/py

*Scopus*

TITLE-ABS-KEY ( ( "laparoscop*" OR "minimally invasive" ) AND ( "liver resection" OR hepatectomy OR "liver surgery" ) AND ( "hepatocellular carcinoma" OR hcc OR "primary liver cancer" ) AND ( cirrhosis OR "liver cirrhosis" OR "chronic liver disease" ) ) AND PUBYEAR < 2021

**FLSTN-AH**

*Pubmed*

(("advanced hepatocellular carcinoma"[Title/Abstract] OR "unresectable hepatocellular carcinoma"[Title/Abstract] OR aHCC[Title/Abstract]) AND (("systemic therapy"[Title/Abstract] OR "first-line therapy"[Title/Abstract] OR immunotherapy[Title/Abstract] OR "targeted therapy"[Title/Abstract]) OR (sorafenib[Title/Abstract] OR atezolizumab[Title/Abstract] OR bevacizumab[Title/Abstract] OR tremelimumab[Title/Abstract] OR durvalumab[Title/Abstract] OR cabozantinib[Title/Abstract] OR nivolumab[Title/Abstract] OR lenvatinib[Title/Abstract] OR linifanib[Title/Abstract] OR brivanib[Title/Abstract] OR sunitinib[Title/Abstract]))) AND ("1900/01/01"[Date - Publication] : "2022/06/07"[Date - Publication])

*Embase*

(('advanced hepatocellular carcinoma':ti,ab OR 'unresectable hepatocellular carcinoma':ti,ab OR aHCC:ti,ab) AND (('systemic therapy':ti,ab OR 'first-line therapy':ti,ab OR immunotherapy:ti,ab OR 'targeted therapy':ti,ab) OR (sorafenib:ti,ab OR atezolizumab:ti,ab OR bevacizumab:ti,ab OR tremelimumab:ti,ab OR durvalumab:ti,ab OR cabozantinib:ti,ab OR nivolumab:ti,ab OR lenvatinib:ti,ab OR linifanib:ti,ab OR brivanib:ti,ab OR sunitinib:ti,ab))) AND [1900-2022]/py

*Scopus*

TITLE-ABS-KEY(("advanced hepatocellular carcinoma" OR "unresectable hepatocellular carcinoma" OR aHCC)) AND TITLE-ABS-KEY(( "systemic therapy" OR "first-line therapy" OR immunotherapy OR "targeted therapy" ) OR ( sorafenib OR atezolizumab OR bevacizumab OR tremelimumab OR durvalumab OR cabozantinib OR nivolumab OR lenvatinib OR linifanib OR brivanib OR sunitinib )) AND PUBYEAR < 2023

*Cochrane*

(hepatocellular carcinoma OR HCC OR "liver cancer") AND (sorafenib OR lenvatinib OR donafenib OR "tyrosine kinase inhibitor" OR "immune checkpoint inhibitor" OR PD-1 OR PD-L1 OR CTLA-4) AND ("randomized controlled trial" OR "phase III")

**TLP-AH**

*Pubmed*

((("Carcinoma, Hepatocellular"[Mesh] OR "Carcinoma, Hepatocellular" OR HCC OR "liver cancer") AND ("Lenvatinib"[Supplementary Concept] OR lenvatinib OR E7080) AND ("Chemoembolization, Therapeutic"[Mesh] OR "chemoembolization, therapeutic" OR TACE) AND (("Programmed Cell Death 1 Receptor"[Mesh] OR "programmed cell death 1 receptor" OR "PD-1 inhibitor" OR "PD-1 blockade" OR "immune checkpoint inhibitor" OR nivolumab OR pembrolizumab OR camrelizumab OR sintilimab OR toripalimab OR tislelizumab))) AND ("1900/01/01"[Date - Publication] : "2023/05/02"[Date - Publication]))

*Embase*

(('hepatocellular carcinoma'/exp OR hepatocellular) AND carcinoma OR hcc OR 'liver cancer') AND ('lenvatinib'/exp OR lenvatinib OR e7080) AND (('transarterial chemoembolization'/exp OR transarterial) AND chemoembolization OR tace) AND ('programmed cell death 1 receptor'/exp OR 'programmed cell death 1 receptor' OR 'pd-1 inhibitor' OR 'immune checkpoint inhibitor' OR nivolumab OR pembrolizumab OR camrelizumab OR sintilimab OR toripalimab OR tislelizumab) AND [1900-01-01]/sd AND to AND [2023-05-02]/sd

*Web of science*

TS=( "Carcinoma, Hepatocellular" OR HCC OR "liver cancer" ) AND TS=( lenvatinib OR E7080 ) AND TS=( "Chemoembolization, Therapeutic" OR TACE OR chemoembolization ) AND TS=( "Programmed Cell Death 1 Receptor" OR "PD-1 inhibitor" OR "immune checkpoint inhibitor" OR nivolumab OR pembrolizumab OR camrelizumab OR sintilimab OR toripalimab OR tislelizumab ) AND PY=(1900-2023)

*Cochrane*

((Carcinoma, Hepatocellular:ti,ab,kw OR HCC OR "liver cancer") AND (lenvatinib:ti,ab,kw OR E7080) AND ( "Chemoembolization, Therapeutic":ti,ab,kw OR TACE OR chemoembolization) AND ("Programmed Cell Death 1 Receptor":ti,ab,kw OR "PD-1 inhibitor":ti,ab,kw OR "immune checkpoint inhibitor":ti,ab,kw OR nivolumab OR pembrolizumab OR camrelizumab OR sintilimab OR toripalimab OR tislelizumab))

**NCN-UH**

*Pubmed*

(("hepatocellular carcinoma"[tiab] OR HCC[tiab] OR "liver cancer"[tiab]) AND (sorafenib[tiab] OR lenvatinib[tiab] OR donafenib[tiab] OR "tyrosine kinase inhibitor"[tiab] OR "immune checkpoint inhibitor"[tiab] OR PD-1[tiab] OR PD-L1[tiab] OR CTLA-4[tiab]) AND ("Randomized Controlled Trial"[Publication Type] OR "Phase III"[tiab]))

*Cochrane*

("hepatocellular carcinoma" OR HCC OR "liver cancer") AND (unresectable OR advanced OR inoperable) AND ((immune NEXT checkpoint NEXT inhibitor*) OR PD-1 OR PD-L1 OR CTLA-4 OR immunotherapy OR (tyrosine NEXT kinase NEXT inhibitor*) OR sorafenib OR lenvatinib OR donafenib) AND ("Phase III" OR "phase 3" OR "randomized controlled trial" OR RCT)

*Embase*

('hepatocellular carcinoma'exp OR hepatocellular carcinoma OR HCC OR liver cancer) AND ('unresectable' OR 'advanced' OR 'inoperable') AND ('immune checkpoint inhibitor' OR 'PD-1' OR 'PD-L1' OR 'CTLA-4' OR 'immunotherapy' OR 'tyrosine kinase inhibitor' OR 'sorafenib' OR 'lenvatinib' OR 'donafenib') AND ('phase III' OR 'phase 3' OR 'randomized controlled trial' OR 'RCT') NOT ('meta analysis' OR 'systematic review' OR 'case report') AND [2007-2022]py AND [english]lim

**FSTN-AH**

*Pubmed*

( ("Carcinoma, Hepatocellular"[Mesh] OR "hepatocellular carcinoma"[tiab] OR HCC[tiab] OR "liver cancer"[tiab]) AND ( "Immunotherapy"[Mesh] OR immunotherapy[tiab] OR "Protein Kinase Inhibitors"[Mesh] OR "tyrosine kinase inhibitor"[tiab] OR TKI[tiab] OR "Immune Checkpoint Inhibitors"[Mesh] OR "immune checkpoint inhibitor"[tiab] OR "Antibodies, Monoclonal"[Mesh] OR "monoclonal antibody"[tiab] OR (sorafenib[tiab] OR lenvatinib[tiab] OR cabozantinib[tiab] OR donafenib[tiab] OR atezolizumab[tiab] OR bevacizumab[tiab] OR pembrolizumab[tiab] OR nivolumab[tiab] OR durvalumab[tiab] OR tremelimumab[tiab] OR sintilimab[tiab] OR camrelizumab[tiab] OR tislelizumab[tiab] OR rivoceranib[tiab]) ) AND (advanced[tiab] OR unresectable[tiab] OR metastatic[tiab]) AND ("2007/01/01"[Date - Publication] : "2022/11/24"[Date - Publication]) )

*Cochrane*

("hepatocellular carcinoma" OR "HCC" OR "liver cancer") AND ("systemic therapy" OR "immunotherapy" OR "tyrosine kinase inhibitor" OR "TKI" OR "immune checkpoint inhibitor" OR "monoclonal antibody") AND ("advanced" OR "unresectable" OR "inoperable")

*Embase*

('hepatocellular carcinoma'/de OR 'hepatocellular carcinoma':ti,ab OR HCC:ti,ab OR 'liver cancer':ti,ab) AND ('immunotherapy'/de OR immunotherapy:ti,ab OR 'tyrosine kinase inhibitor'/de OR 'tyrosine kinase inhibitor':ti,ab OR TKI:ti,ab OR 'immune checkpoint inhibitor'/de OR 'immune checkpoint inhibitor':ti,ab OR 'monoclonal antibody':ti,ab) AND (advanced:ti,ab OR unresectable:ti,ab OR inoperable:ti,ab) AND [2007-2022]/py

**PTICI-PTHO**

*Pubmed*

(("Carcinoma, Hepatocellular"[Mesh] OR "hepatocellular carcinoma"[Title/Abstract] OR HCC[Title/Abstract] OR "hepatic cancer"[Title/Abstract] OR "liver cancer"[Title/Abstract] OR "liver neoplasm"[Title/Abstract] OR "hepatic malignancy"[Title/Abstract]) AND ("Liver Transplantation"[Mesh] OR "liver transplantation"[Title/Abstract] OR "liver transplant"[Title/Abstract] OR OLT[Title/Abstract] OR "orthotopic liver transplantation"[Title/Abstract]) AND ("Immune Checkpoint Inhibitors"[Mesh] OR "immune checkpoint inhibitor"[Title/Abstract] OR "immune checkpoint blockade"[Title/Abstract] OR ICI[Title/Abstract] OR "PD-1 inhibitor"[Title/Abstract] OR "PD-L1 inhibitor"[Title/Abstract] OR "CTLA-4 inhibitor"[Title/Abstract] OR "programmed cell death 1"[Title/Abstract] OR "programmed cell death ligand 1"[Title/Abstract] OR nivolumab[Title/Abstract] OR pembrolizumab[Title/Abstract] OR atezolizumab[Title/Abstract] OR sintilimab[Title/Abstract] OR camrelizumab[Title/Abstract] OR durvalumab[Title/Abstract] OR ipilimumab[Title/Abstract] OR tremelimumab[Title/Abstract])) AND ("2000/01/01"[PDAT] : "2024/01/13"[PDAT])

*Web of science*

TS=(("hepatocellular carcinoma" OR HCC OR "hepatic cancer" OR "liver cancer" OR "liver neoplasm" OR "hepatic malignancy") AND ("liver transplantation" OR "liver transplant" OR OLT OR "orthotopic liver transplantation") AND ("immune checkpoint inhibitor" OR ICI OR "immune checkpoint blockade" OR "PD-1 inhibitor" OR "PD-L1 inhibitor" OR "CTLA-4 inhibitor" OR "programmed cell death 1" OR "programmed cell death ligand 1" OR nivolumab OR pembrolizumab OR atezolizumab OR sintilimab OR camrelizumab OR durvalumab OR ipilimumab OR tremelimumab))

*Scopus*

TITLE-ABS-KEY(("hepatocellular carcinoma" OR HCC OR "hepatic cancer" OR "liver cancer" OR "liver neoplasm" OR "hepatic malignancy") AND ("liver transplantation" OR "liver transplant" OR OLT OR "orthotopic liver transplantation") AND ("immune checkpoint inhibitor" OR ICI OR "immune checkpoint blockade" OR "PD-1 inhibitor" OR "PD-L1 inhibitor" OR "CTLA-4 inhibitor" OR "programmed cell death 1" OR "programmed cell death ligand 1" OR nivolumab OR pembrolizumab OR atezolizumab OR sintilimab OR camrelizumab OR durvalumab OR ipilimumab OR tremelimumab)) AND PUBYEAR > 1999 AND PUBYEAR < 2025

**SBRT-HMG**

*Pubmed*

("Carcinoma, Hepatocellular"[Mesh] OR hepatocellular carcinoma[tiab] OR HCC[tiab] OR primary liver cancer[tiab]) AND (stereotactic body radiotherapy[tiab] OR SBRT[tiab] OR stereotactic ablative radiotherapy[tiab] OR SABR[tiab]) AND ("2003/01/01"[PDAT] : "2022/10/31"[PDAT])

*Embase*

('hepatocellular carcinoma'/exp OR hepatocellular carcinoma:ti,ab OR HCC:ti,ab OR primary liver cancer:ti,ab) AND ('stereotactic body radiotherapy'/exp OR stereotactic body radiotherapy:ti,ab OR SBRT:ti,ab OR stereotactic ablative radiotherapy:ti,ab OR SABR:ti,ab) AND [2003-2022]/py

*Scopus*

TITLE-ABS-KEY("hepatocellular carcinoma" OR HCC OR "primary liver cancer") AND TITLE-ABS-KEY("stereotactic body radiotherapy" OR SBRT OR "stereotactic ablative radiotherapy" OR SABR) AND PUBYEAR > 2002 AND PUBYEAR < 2023

*Cochrane*

((hepatocellular carcinoma OR HCC OR "primary liver cancer") AND (stereotactic body radiotherapy OR SBRT OR "stereotactic ablative radiotherapy" OR SABR))

**STSN-AH**

*Pubmed*

("Carcinoma, Hepatocellular"[Mesh] OR hepatocellular carcinoma[tiab] OR HCC[tiab] OR primary liver cancer[tiab]) AND (stereotactic body radiotherapy[tiab] OR SBRT[tiab] OR stereotactic ablative radiotherapy[tiab] OR SABR[tiab]) AND ("2003/01/01"[PDAT] : "2022/10/31"[PDAT])

*Embase*

('hepatocellular carcinoma'/exp OR hepatocellular carcinoma:ti,ab OR HCC:ti,ab OR primary liver cancer:ti,ab) AND ('stereotactic body radiotherapy'/exp OR stereotactic body radiotherapy:ti,ab OR SBRT:ti,ab OR stereotactic ablative radiotherapy:ti,ab OR SABR:ti,ab) AND [2003-2022]/py

*Scopus*

TITLE-ABS-KEY("hepatocellular carcinoma" OR HCC OR "primary liver cancer") AND TITLE-ABS-KEY("stereotactic body radiotherapy" OR SBRT OR "stereotactic ablative radiotherapy" OR SABR) AND PUBYEAR > 2002 AND PUBYEAR < 2023

*Cochrane*

((hepatocellular carcinoma OR HCC OR "primary liver cancer") AND (stereotactic body radiotherapy OR SBRT OR "stereotactic ablative radiotherapy" OR SABR))

**LRAMC**

*Pubmed*

("Carcinoma, Hepatocellular"[Mesh] OR (hepatocellular[tiab] AND carcinoma[tiab]) OR hepatocarcinoma[tiab] OR "liver cell carcinoma"[tiab]) AND ("Hepatectomy"[Mesh] OR hepatectomy[tiab] OR "hepatic resection"[tiab] OR "surgical resection"[tiab]) AND ("Radiofrequency Ablation"[Mesh] OR "radiofrequency ablation"[tiab] OR RFA[tiab]) AND ("1900/01/01"[Date - Publication] : "2020/03/01"[Date - Publication])

*Embase*

('liver cell carcinoma'/exp OR 'liver cell carcinoma':ab,ti OR 'hepatocellular carcinoma':ab,ti OR 'liver cell cancer':ab,ti OR hepatocarcinoma:ab,ti OR 'primary liver cell cancer':ab,ti) AND ('liver resection'/exp OR hepatectomy:ab,ti OR 'liver resection':ab,ti OR 'hepatic resection':ab,ti OR 'surgical resection':ab,ti OR 'liver surgery':ab,ti) AND ('radiofrequency ablation'/exp OR 'radiofrequency ablation':ab,ti OR rfa:ab,ti OR ablation:ab,ti OR radiofrequency:ab,ti) AND [1950-2020]/py

*Cochrane*

(MeSH DESCRIPTOR Carcinoma, Hepatocellular EXPLODE ALL TREES OR "hepatoma":TI,AB,KW OR "liver cell carcinoma":TI,AB,KW OR "liver cell cancer":TI,AB,KW OR "hepatocellular carcinoma":TI,AB,KW OR "hepatocarcinoma":TI,AB,KW) AND (MeSH DESCRIPTOR Hepatectomy EXPLODE ALL TREES OR "hepatic resection":TI,AB,KW OR "liver resection":TI,AB,KW OR "hepatectomy":TI,AB,KW OR "surgical resection":TI,AB,KW) AND (MeSH DESCRIPTOR Catheter Ablation EXPLODE ALL TREES OR "radiofrequency ablation":TI,AB,KW OR "RFA":TI,AB,KW OR "radiofrequency":TI,AB,KW OR "ablation":TI,AB,KW)

## A9 Comparative test results of kappa and PABAK

**Normality Tests (Shapiro-Wilk)**

Kappa: W = 0.9158937 , p-value = 0.3593284

PABAK: W = 0.9365802 , p-value = 0.5463969

**Paired Tests**

t-test: t = -11.74041 , p-value = 2.532224e-06

Wilcoxon test: V = 0 , p-value = 0.00390625

Effect size (Cohen's d) = -3.715635

**Descriptive Statistics**

Kappa: mean = 0.2581667 , sd = 0.1861662

PABAK: mean = 0.8729333 , sd = 0.08044453

**Conclusion**

Better metric: PABAK

Based on: t-test

Data normality: Normal

# Supplementary Tables

Table S1 The screening status of the number of literature from various datasets by the analysis system

| Dataset | Number of records system included  (n1=1338) | Number of records actually included  (n2=129) | Number of records initially identified (N=27982) |
| --- | --- | --- | --- |
| LORC-CH | 42 | 11 | 770 |
| FLSTN-AH | 522 | 11 | 5417 |
| TLP-AH | 39 | 12 | 309 |
| NCN-UH | 161 | 9 | 1698 |
| FSTN-AH | 133 | 15 | 8733 |
| PTICI-PTHO | 34 | 21 | 558 |
| SBRT-HMG | 114 | 13 | 897 |
| STSN-AH | 250 | 13 | 3522 |
| LRAMC | 43 | 24 | 6078 |

Table S2 The performance of the analysis system across various datasets in the exclusion and inclusion target

| Dataset | Accuracy | Precision  (Excluded) | Recall  (Excluded) | F1 score  (Excluded) | Precision  (Included) | Recall  (Included) | F1 score  (Included) | Kappa | PABAK |
| --- | --- | --- | --- | --- | --- | --- | --- | --- | --- |
| LORC-CH | 0.9597 | 1.0000 | 0.9592 | 0.9792 | 0.2619 | 1.0000 | 0.4151 | 0.4015 | 0.9195 |
| FLSTN-AH | 0.9057 | 1.0000 | 0.9055 | 0.9504 | 0.0211 | 1.0000 | 0.0413 | 0.0374 | 0.8113 |
| TLP-AH | 0.8997 | 0.9926 | 0.9024 | 0.9453 | 0.2564 | 0.8333 | 0.3922 | 0.3538 | 0.7994 |
| NCN-UH | 0.9105 | 1.0000 | 0.9100 | 0.9529 | 0.0559 | 1.0000 | 0.1059 | 0.0968 | 0.8210 |
| FSTN-AH | 0.9865 | 1.0000 | 0.9865 | 0.9932 | 0.1128 | 1.0000 | 0.2027 | 0.2002 | 0.9730 |
| PTICI-PTHO | 0.9588 | 0.9905 | 0.9665 | 0.9783 | 0.4706 | 0.7619 | 0.5818 | 0.5614 | 0.9176 |
| SBRT-HMG | 0.8807 | 0.9962 | 0.8824 | 0.9358 | 0.0877 | 0.7692 | 0.1575 | 0.1350 | 0.7614 |
| STSN-AH | 0.9327 | 1.0000 | 0.9325 | 0.9650 | 0.0520 | 1.0000 | 0.0989 | 0.0925 | 0.8654 |
| LRAMC | 0.9939 | 0.9985 | 0.9954 | 0.9969 | 0.3488 | 0.6250 | 0.4478 | 0.4449 | 0.9878 |
| Total average  (weighted; mean ± SD) | 0.9554 ± 0.0388 | 0.9993 ± 0.0017 | 0.9559 ± 0.0391 | 0.9767 ± 0.0206 | 0.1472 ± 0.1289 | 0.9046 ± 0.1568 | 0.2198 ± 0.1559 | 0.2144 ± 0.1554 | 0.9109 ± 0.0776 |
| Total average  (macro; mean ± SD) | 0.9365 ± 0.0402 | 0.9975 ± 0.0037 | 0.9378 ± 0.0405 | 0.9663 ± 0.0217 | 0.1852 ± 0.1562 | 0.8877 ± 0.1436 | 0.2715 ± 0.1906 | 0.2582 ± 0.1862 | 0.8729 ± 0.0804 |

Table S3 Performance of imbalanced statistical metrics across nine datasets

| Dataset | Balanced accuracy | Matthews Correlation Coefficient | F-β (β=2) |
| --- | --- | --- | --- |
| LORC-CH | 0.9796 | 0.5012 | 0.6395 |
| FLSTN-AH | 0.9528 | 0.1381 | 0.0973 |
| TLP-AH | 0.8679 | 0.4280 | 0.5747 |
| NCN-UH | 0.9550 | 0.2255 | 0.2284 |
| FSTN-AH | 0.9933 | 0.3335 | 0.3886 |
| PTICI-PTHO | 0.8642 | 0.5795 | 0.6780 |
| SBRT-HMG | 0.8258 | 0.2338 | 0.3012 |
| STSN-AH | 0.9663 | 0.2202 | 0.2152 |
| LRAMC | 0.8102 | 0.4642 | 0.5396 |
| Total average (weighted) | 0.9302 | 0.2732 | 0.4459 |
| Total average (macro) | 0.9128 | 0.2677 | 0.5048 |

Table S4 Platform unit prices and estimated analysis costs for each model

| Model label | Model name | Input cost ($/1kT) ^a^ | Output cost ($/1kT) ^a^ | Estimated cost ($/1k entries) ^a, b^ |
| --- | --- | --- | --- | --- |
| Model A | Doubao-1.5-pro-32k | 0.0001 | 0.0003 | 0.0948 |
| Model B | DeepSeek-v3 | 0.0003 | 0.0011 | 0.3193 |
| Model C | DeepSeek-R1-Distill-Qwen-7B | 0.0001 | 0.0003 | ~0.1000 |

^a^ This price is derived by converting the benchmark pricing of the model from Volcano Engine (a cloud computing platform operating in Mainland China) with reference to the USD exchange rate.

^b^ The estimated cost is based on the actual execution costs across various datasets. Due to differences in the number of fields processed in practice, this estimated cost may vary.

Table S5 Detailed time and financial costs of the three large language model-based analytical system across nine datasets

| Dataset | Sample size | Temporal costs (seconds) | | | | Economic costs (USD) | | | |
| --- | --- | --- | --- | --- | --- | --- | --- | --- | --- |
|  |  | Model A | Model B | Model C | Total | Model A | Model B | Model C | Total |
| LORC-CH | 770 | 248.2 | 349.4 | 0.0 | 597.6 | 0.0708 | 0.2431 | 0.0000 | 0.3139 |
| FLSTN-AH | 5417 | 1796.9 | 1945.4 | 14.0 | 3756.3 | 0.5233 | 1.7484 | 0.0005 | 2.2721 |
| TLP-AH | 309 | 109.7 | 129.6 | 9.1 | 248.4 | 0.0316 | 0.1035 | 0.0002 | 0.1353 |
| NCN-UH | 1698 | 560.1 | 606.7 | 11.9 | 1178.7 | 0.1679 | 0.5597 | 0.0002 | 0.7278 |
| FSTN-AH | 8733 | 2842.9 | 2995.3 | 12.0 | 5850.2 | 0.8407 | 2.8359 | 0.0001 | 3.6768 |
| PTICI-PTHO | 558 | 190.8 | 197.1 | 0.0 | 387.9 | 0.0444 | 0.1572 | 0.0000 | 0.2015 |
| SBRT-HMG | 897 | 313.6 | 341.5 | 8.3 | 663.4 | 0.0865 | 0.2858 | 0.0002 | 0.3725 |
| STSN-AH | 3522 | 1309.0 | 1312.0 | 0.0 | 2621.0 | 0.3617 | 1.1863 | 0.0000 | 1.5480 |
| LRAMC | 6078 | 2150.5 | 2220.2 | 0.0 | 4370.7 | 0.5258 | 1.8147 | 0.0000 | 2.3405 |
| Total | 27982 | 9521.7 | 10097.2 | 55.3 | 19674.2 | 2.6527 | 8.9346 | 0.0012 | 11.5885 |
| Average (mean ± SD) | 3109 | 1058.0 ± 1006.1 | 1121.9 ± 1045.3 | 6.1 ± 6.1 | 2186.0 ± 2052.4 | 0.2947 ± 0.2850 | 0.9927 ± 0.9630 | 0.0001 ± 0.0001 | 1.2876 ± 1.2480 |
| Cost per 1k entries ^a^ | 1000 | 340.3 | 360.8 | 2.0 | 703.1 | 0.0948 | 0.3193 | ~0.0000 | 0.4141 |

^a^ The "per 1k entries" refers to every thousand pieces of literature data that are initially simulated to enter the system. Model C is capable of handling literature entries where Model A and Model B have conflicting opinions. Therefore, the cost per 1k entries associated with Model C refers to the processing cost of the conflicting entries within 1k initial entries.

Table S6 Reversion data during the analysis of Model B

| Dataset | Total records | Total reversions | Correct reversions (Fixed) | Incorrect reversions (Broke) |
| --- | --- | --- | --- | --- |
| LORC-CH | 770 | 0 | 0 | 0 |
| FLSTN-AH | 5417 | 4 | 3 | 1 |
| TLP-AH | 309 | 1 | 0 | 1 |
| NCN-UH | 1698 | 1 | 1 | 0 |
| FSTN-AH | 8733 | 1 | 0 | 1 |
| PTICI-PTHO | 558 | 0 | 0 | 0 |
| SBRT-HMG | 897 | 2 | 1 | 1 |
| STSN-AH | 3522 | 0 | 0 | 0 |
| LRAMC | 6078 | 0 | 0 | 0 |
| Overall Summary | 27982 | 9 | 5 | 4 |

# Supplementary Figures


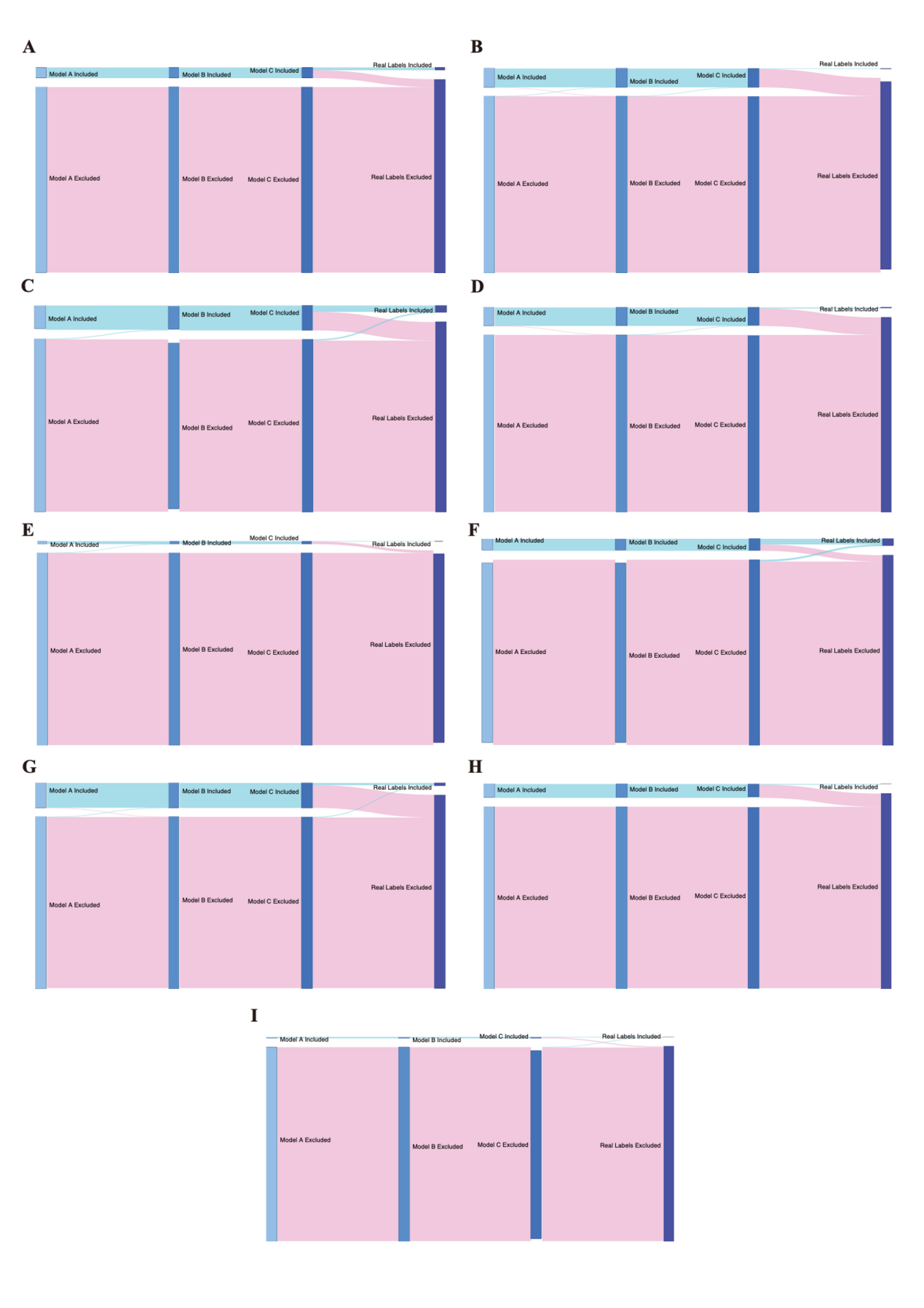


Figure S1 Information flow of LLMs in literature screening on nine datasets.

The three blue columns from left to right represent the decisions of Model A, Model B, and Model C. The dark blue column on the far right represents the actual inclusion exclusion. The red streamline means that in the next model, the entry is classified into the exclusion category. The blue streamline means that in the next model, the entry is classified into the inclusion category. All streamlines are based on (A) LORC-CH: laparoscopic versus open resection in cirrhotic hepatocellular carcinoma; (B) FLSTN-AH: first-line systemic therapy network meta-analysis for advanced hepatocellular carcinoma; (C) TLP-AH: transarterial chemoembolization plus lenvatinib plus programmed death receptor 1 inhibitor in advanced hepatocellular carcinoma; (D) NCN-UH: novel combination network meta-analysis for unresectable hepatocellular carcinoma; (E) FSTN-AH: frontline systemic therapy network meta-analysis for advanced hepatocellular carcinoma; (F) PTICI-PTHO: pre-transplant immune checkpoint inhibitors impact on post-transplant hepatocellular carcinoma outcomes; (G) SBRT-HMG: stereotactic body radiation therapy in hepatocellular carcinoma meta-analysis and guidelines; (H) STSN-AH: systemic treatment sequencing network meta-analysis for advanced hepatocellular carcinoma; (I) LRAMC: liver resection versus ablation within Milan criteria.
